# Coordinated loading of IRG resistance GTPases on to the *Toxoplasma gondii* parasitophorous vacuole

**DOI:** 10.1111/j.1462-5822.2010.01443.x

**Published:** 2010-03-04

**Authors:** Aliaksandr Khaminets, Julia P Hunn, Stephanie Könen-Waisman, Yang O Zhao, Daniela Preukschat, Jörn Coers, Jon P Boyle, Yi-Ching Ong, John C Boothroyd, Gabriela Reichmann, Jonathan C Howard

**Affiliations:** 1 Institute for Genetics, University of Cologne, Zülpicher StrasseCologne 50674, Germany; 2 Department of Microbiology, Harvard University Medical SchoolCambridge, MA 02115, USA; 3 Department of Biological Sciences, University of PittsburghPittsburgh, PA 15260, USA; 4 Department of Microbiology and Immunology, Stanford UniversityStanford, CA 94305, USA; 5 Institute for Medical Microbiology, University of DüsseldorfDüsseldorf 40225, Germany

## Abstract

The immunity-related GTPases (IRGs) constitute an interferon-induced intracellular resistance mechanism in mice against *Toxoplasma gondii*. IRG proteins accumulate on the parasitophorous vacuole membrane (PVM), leading to its disruption and to death of the parasite. How IRGs target the PVM is unknown. We show that accumulation of IRGs on the PVM begins minutes after parasite invasion and increases for about 1 h. Targeting occurs independently of several signalling pathways and the microtubule network, suggesting that IRG transport is diffusion-driven. The intensity of IRG accumulation on the PVM, however, is reduced in absence of the autophagy regulator, Atg5. In wild-type cells IRG proteins accumulate cooperatively on PVMs in a definite order reflecting a temporal hierarchy, with Irgb6 and Irgb10 apparently acting as pioneers. Loading of IRG proteins onto the vacuoles of virulent *Toxoplasma* strains is attenuated and the two pioneer IRGs are the most affected. The polymorphic rhoptry kinases, ROP16, ROP18 and the catalytically inactive proteins, ROP5A–D, are not individually responsible for this effect. Thus IRG proteins protect mice against avirulent strains of *Toxoplasma* but fail against virulent strains. The complex cooperative behaviour of IRG proteins in resisting *Toxoplasma* may hint at undiscovered complexity also in virulence mechanisms.

## Introduction

Mice deficient in single members of the large family of IFN-inducible immunity-related GTPases (IRG proteins or p47 GTPases) are vulnerable to a number of intracellular pathogens [reviewed in Taylor (2004; 2007;); MacMicking (2005); Martens and Howard (2006)]. The most striking effects have been seen after infection with avirulent strains of *Toxoplasma gondii*, where to date five IRG proteins have been individually implicated in resistance (Halonen *et al.*, 2001; Butcher *et al.*, 2005; Martens *et al.*, 2005; Ling *et al.*, 2006; Zhao *et al.*, 2009a). There is no consensus about how IRG proteins exercise their resistance function (Howard, 2008). They have been implicated in rapid maturation of the *Mycobacterium tuberculosis* phagosome (MacMicking *et al.*, 2003), in the initiation of an autophagic process in mycobacterial infection (Gutierrez *et al.*, 2004), and most relevantly to the present paper, in the direct disruption of the *T. gondii* parasitophorous vacuole membrane (PVM) (Martens *et al.*, 2005; Ling *et al.*, 2006; Melzer *et al.*, 2008; Zhao *et al.*, 2008; 2009a;).

IRG proteins are strongly induced in many cell types by exposure to IFNγ (Boehm *et al.*, 1998). Protein expression can be detected in mouse embryonic fibroblasts (MEFs) 3 h after induction (Boehm, 1999) and reaches a plateau between 24 and 48 h. The proteins distribute among distinct cellular compartments, Irgm1 to the Golgi (Martens *et al.*, 2004) and the endolysosomal compartment (Zhao, 2008; Zhao *et al.*, 2010), Irgm2 to the Golgi (Martens and Howard, 2006; Hunn *et al.*, 2008), Irgm3 (Taylor *et al.*, 1997) and Irga6 (Zerrahn *et al.*, 2002; Martens *et al.*, 2004) to the endoplasmic reticulum (ER). Native Irgb6 and Irgd are predominantly or wholly cytosolic (Martens *et al.*, 2004), Irga6 partitions roughly 60:40 between ER membrane and cytosol, whileIrgm1, Irgm2 and Irgm3 are largely or exclusively membrane-bound (Boehm *et al.*, 1998; Martens *et al.*, 2004; Martens and Howard, 2006). After infection of IFNγ-induced cells with avirulent *T. gondii*, five out of the six IRG proteins studied were found in high density on the PVM (Martens *et al.*, 2005). We show here that Irgb10 is also found on the *T. gondii* PVM. Irgm1 has never been reported on the PVM, but accumulates on phagocytic cups and phagosomes in cells phagocytosing *M. tuberculosis* or latex beads (Butcher *et al.*, 2005; Martens *et al.*, 2005; Shenoy *et al.*, 2007).

It was initially shown in IFNγ-induced primary mouse astrocytes that the *T. gondii* vacuolar membrane becomes ruffled, vesiculated and ultimately disrupted, exposing the parasite to the cytoplasm, a process dependent on the presence of IRG proteins (Martens *et al.*, 2005), a process since confirmed in mouse astrocytes (Melzer *et al.*, 2008) and peritoneal (Ling *et al.*, 2006) and bone-marrow-derived (Zhao *et al.*, 2008) macrophages and MEFs (Zhao *et al.*, 2009a). Thus the disruption process is not cell type-specific. Recent experiments (Zhao *et al.*, 2009a,b;) strongly suggest that disruption of the IRG-loaded PV is the critical step in *T. gondii* restriction in IFNγ-induced cells. Disruption of the PV is invariably followed by the death of the parasite and shortly thereafter by the necrotic death of the infected cell (Zhao *et al.*, 2009a). The programme is complete within a few hours of infection and before tachyzoite replication. Virulent *T. gondii* strains, however, are not efficiently restricted by IFNγ induction, correlated with reduced or absent vacuolar disruption and minimal death of the parasites and host cells (Zhao *et al.*, 2009a). The reduced disruption of vacuoles containing virulent *T. gondii* is itself correlated with deficient loading of IRG proteins onto the vacuolar membrane (Zhao *et al.*, 2009b,c;).

The process by which IRG proteins access the *T. gondii* PVM has not previously been subjected to systematic analysis, despite its relevance to the resistance mechanism. We show that loading of IRG proteins onto the PVM begins almost immediately after infection, and rises rapidly for about 1 h. There is a surprising heterogeneity in the intensity of vacuolar loading of IRG proteins, with some vacuoles remaining either completely or almost completely un-loaded. IRG proteins load onto the vacuole in a complex pattern, with Irgb6 and Irgb10 being the most efficient and also the first to load. We seek to explain how IRG proteins make their way from their normal cytoplasmic compartments to the vacuole, and conclude that diffusion from the cytosolic pools, rather than active transport, is the most likely route. Our results confirm a recent report that the autophagic regulator, Atg5, is required for normal loading and suggest that the effect is due to dysregulation of the GTPase cycle of the GKS subset of IRG proteins. Finally, we show that the pattern of vacuolar loading is determined by the strain of *T. gondii* that inhabits the vacuole. The great majority of PVMs of type I virulent strains are far less intensely loaded with IRG proteins than those of type II or type III avirulent strains in single as well as in co-infections. Because the restriction of virulent strains by IFN-treated cells is far less efficient than the restriction of avirulent strains, the association with vacuolar IRG loading confirms the importance of IRG proteins in resistance of mice to *T. gondii*, and suggests that they act at the PVM itself. We show that the virulence-associated polymorphic *T. gondii* kinases, ROP16 and ROP18, as well as a virulence-associated genomic segment containing four related pseudokinases, ROP5A–D, are not individually responsible for virulence-related effect on IRG loading.

## Results

### Rapid loading of the *T. gondii* ME49 PVM with IRG proteins in MEFs

**Fig. 1:**
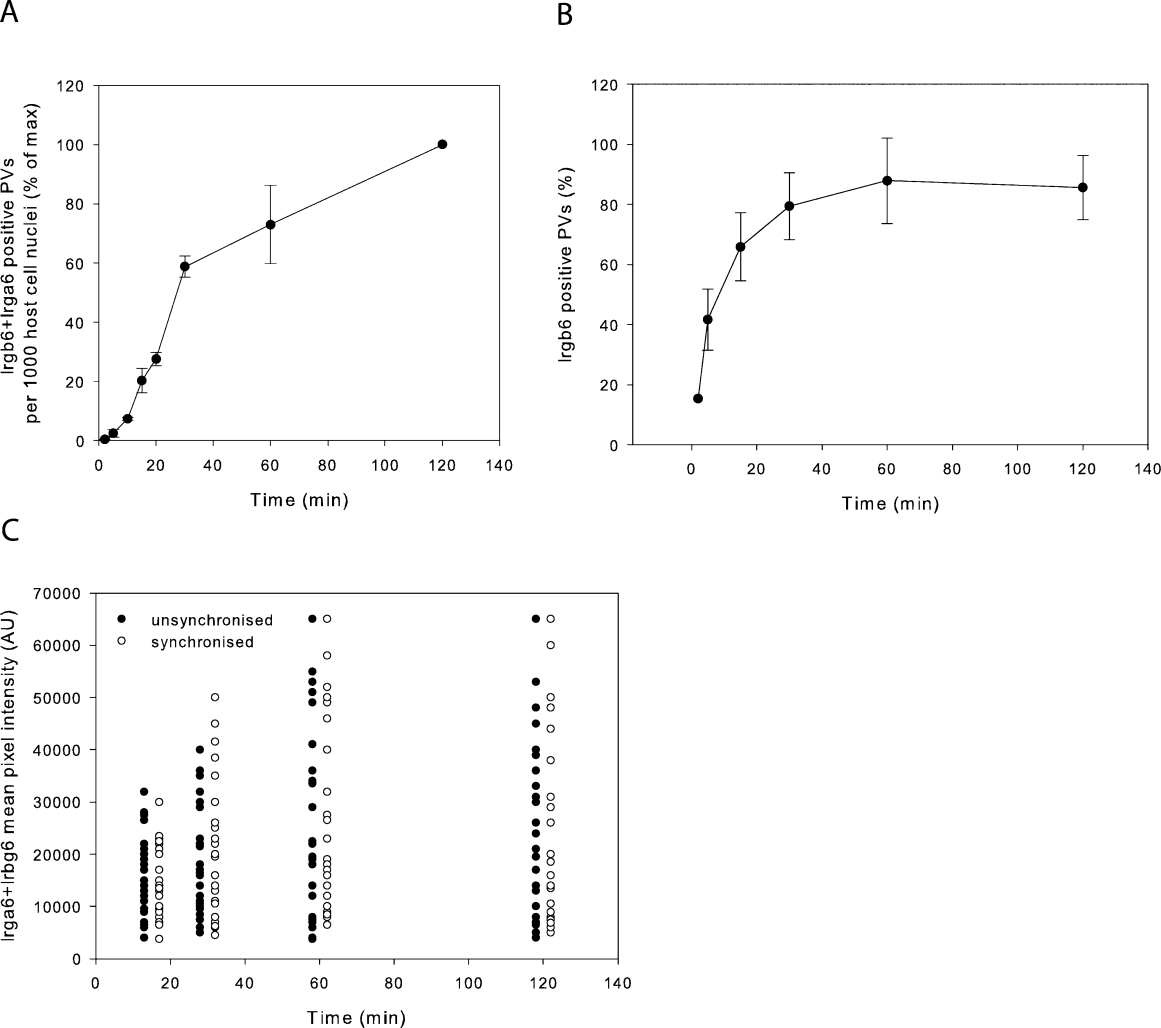
Time-course of Irga6 and Irgb6 association with *T. gondii* ME49 PVs. IFNγ-induced C57BL/6 MEFs were infected with *T. gondii* ME49 strain as described in *Experimental procedures*. At intervals from 2.5 min to 2 h after infection slides were prepared for staining simultaneously with antibody reagents against Irga6 (mAb 10D7) and Irgb6 (serum A20) using secondary antibodies coupled with the same fluorochrome to enhance the visible signal (A, C) or against Irgb6 (serum A20) alone (B). DAPI was used to stain the nuclei. A. Loading of IRG proteins begins early after cell penetration. Vacuoles with visible accumulations of IRG proteins on the PVM were counted per 1000 host cell nuclei at each time point and presented as a percentage of maximum. The mean of two independent repetitions and the range between them are shown. B. The frequency of Irgb6-positive vacuoles increased with time after infection. In two independent experiments Irgb6-positive PVs were counted out of 10–100 intracellular parasites at different time points after infection. Mean and ranges are given. The 2 min time point was assayed in only one experiment. C. IRG signal intensity at the PVM increased with time after infection. Fluorescent signal intensities of IRG protein (Irgb6 plus Irga6) on individual vacuoles were measured as described in *Experimental procedures* (see also Fig. S1) at the times indicated. Neither signal intensities nor heterogeneity were detectably affected by synchronized infection (as described in *Experimental procedures*) and thorough removal of free parasites by washing. Open circles: infection was synchronized and free parasites were washed off; closed circles: infection was not synchronized and free parasites were not washed off after inoculation. Twenty-five positive vacuoles were measured at each time point.

IFNγ-induced primary C57BL/6 MEFs were examined for IRG signals at the PVM from 2.5 min up to 2 h after addition of avirulent ME49 *T. gondii* (Fig. 1A). To increase sensitivity, cells were stained simultaneously with antisera against Irga6 and Irgb6 and detected together. The fastest rise in positive vacuoles occurred 15–30 min after infection, but a few vacuoles were detectably loaded with IRG proteins after 2.5 min. Similar results were obtained when the kinetics of Irgb6 loading were analysed separately as a proportion of IRG-positive PVs over infection time with ME49 *T. gondii* (Fig. 1B). The increase in number of labelled vacuoles was accompanied by increased intensity of the signals measured at the PVM (Fig. 1C, filled circles and Fig. S1). There was large variation in the intensity of labelling of individual vacuoles that was not diminished when *T. gondii* infection was synchronized (as described in *Experimental procedures*) and free parasites were washed off after co-incubation with the target cells (Fig. 1C, open circles).

**Fig. 2:**
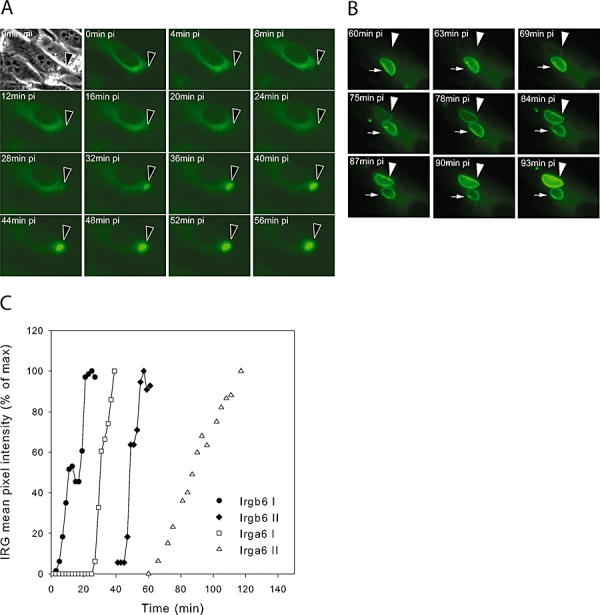
Loading of individual vacuoles by Irga6-ctag1-EGFP or Irgb6-FLAG-EGFP observed by time-lapse microscopy. C57BL/6 MEFs were transfected with the expression plasmid pEGFP-N3-Irga6-ctag1 or pEGFP-N3-Irgb6-FLAG and simultaneously induced with IFNγ. After 24 h, the cells were infected with *T. gondii* ME49 strain in microscope slide chambers as described in Zhao *et al.* (2009a). Cells were observed continuously in order to document the entry of individual parasites and the subsequent accumulation of Irga6-ctag1-EGFP or Irgb6-FLAG-EGFP on the PV. A. and B. Selected frames of two time-lapse videos of Irga6-ctag1-EGFP loading on ME49 *T. gondii* PV. Arrowheads indicate the location of the analysed *T. gondii* PVs. The arrow in (B) indicates a *T. gondii* vacuole already loaded with Irga6-ctag1-EGFP before the initiation of the movie (see also text). [Note that the frames shown in (B) are not a regular time series as some frames were out of focus and have not been included]. The videos from which frames in (A and B) were extracted are presented as Videos S1 and S2 respectively. pi, post inoculation. C. Mean pixel intensities of Irga6 and Irgb6 at the PVM were measured from the vacuoles shown in (A and B) (Irga6 I and Irga6 II respectively) and from two further videos of Irgb6 (Irgb6 I and Irgb6 II; these frames are shown in Fig. S1D), and plotted as percentage of the maximum intensity. The origin on the time axis is the time of addition of *T. gondii* to the cells. The first symbol of each plot gives the time when the observed parasite was seen to enter the cell. In the case of the Irgb6 I movie the protein signal slightly decreased after 13 min because of focus drift on the 15 and 17 min frames and resumed its rise after correction.

The rising phase of both the frequency and intensity data may be attributed to variation in the delay associated with loading of individual vacuoles and indeed to some residual asynchrony in the infection. We therefore used time-lapse photography to observe the loading of a transfected EGFP-tagged version of Irga6 (Irga6-ctag1-EGFP) (Zhao *et al.*, 2009a) on to individual PVMs. The time delays before detecting loading were, respectively, 28 min and 9 min in the two frame series shown in Fig. 2A and B (see also Videos S1 and S2). The signal intensities of Irga6-ctag1-EGFP at the PVM in these videos were quantified in consecutive frames (Fig. 2C). Frames from two further videos showing the accumulation of Irgb6-FLAG-EGFP (Fig. S1D) were also quantified (Fig. 2C, Irgb6 I and II). The four data sets illustrate two independent contributions to the rise in loaded vacuoles shown in Fig. 1. First, there is variation in the time of entry of the *T. gondii* into the cell, which can be as early as the first minute or two in the Irga6 I series (Fig. 2A) but also as late as 60 min in the Irga6 II series (Fig. 2B). Second, there is heterogeneity in the length of the delay after infection before loading begins, which can be as short as 2.5 min in rare vacuoles seen in the fixed preparations (Fig. 1), and in one of the quantified Irgb6 videos (Fig. 2C, Irgb6 I), 9 min in Irga6 II (Fig. 2B and C) and as much as 28 min in Irga6 I (Fig. 2A and C). Once IRG accumulation is initiated it rises roughly linearly for 30–60 min.

### Heterogeneity of *T. gondii* PVM loading with IRG proteins

**Fig. 3:**
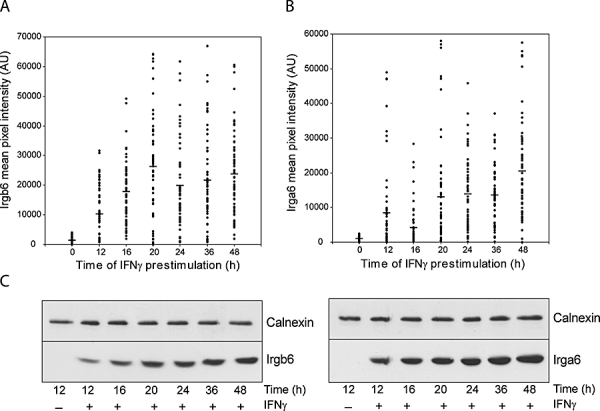
Influence of duration of IFNγ induction on Irga6 and Irgb6 protein levels and on vacuolar loading. MEFs were induced for different times with IFNγ before infection with *T. gondii* strain ME49 for 2 h and stained in immunofluorescence against Irga6 and Irgb6. Co-staining against GRA7 was used to determine intracellular parasites. A. and B. The pixel intensities of (A) Irgb6 (serum A20) and (B) Irga6 (serum 165) signals at the PVM of ME49 vacuoles were determined as described in *Experimental procedures* (see also Fig. S1) and displayed as a function of IFNγ induction time. Sixty PVs were quantified per time point and the arithmetic means are given as horizontal lines. C. In parallel sample cell lysates from MEFs induced for the indicated times with IFNγ were analysed by Western blot for Irga6 (mAb 10D7) and Irgb6 (mAb B34) expression level relative to calnexin as a loading control.

The combined intensities of vacuolar accumulation recorded for Irga6 and Irgb6 during the first 2 h after infection were remarkably heterogeneous (Fig. 1C). We analysed pixel intensities of Irga6 and Irgb6 independently at the PVM in MEFs induced for 12–48 h with IFN and infected for a further 2 h with *T. gondii* (Fig. 3A and B). Absolute levels of Irgb6 and to a lesser extent Irga6 protein in the cells increased with time after IFNγ stimulation (Fig. 3C). Sixty intracellular vacuoles were quantified per time point for Irga6 and Irgb6. The great majority of vacuoles accumulated some IRG protein but the amount accumulated varied from very high values all the way down to the visible threshold and below. The mean expression of Irga6 and Irgb6 increased up to 48 h after IFN induction (Fig. 3C) as did the IRG protein level on the 2 h post-infection PVM (Fig. 3A and B), suggesting that IRG protein accumulation on the vacuole is partly concentration-driven. So heterogeneity in IRG protein level in individual cells could contribute to heterogeneity of PVM loading. Nevertheless most of the observed heterogeneity is evidently intrinsic to the individual vacuole. This could be visualized directly for the cell shown in Fig. 2B (Video S2), which is already infected with one *T. gondii* (arrow) before a second one enters (arrowhead). The PVM of the first parasite already has clear Irga6-ctag1-EGFP accumulation that does not change during the video while the accumulation on the PVM of the second *T. gondii* rapidly overhauls the first one and becomes very bright. Some vacuoles remained free of IRG protein for hours: other vacuoles in the same cell could be heavily loaded. The nature of this vacuole-specific heterogeneity in IRG loading is obviously of interest as it relates directly to the ability of individual parasites to escape attack by IRG resistance proteins.

### Vacuolar loading of IRG proteins is independent of major signalling systems and of microtubules

**Fig. 4:**
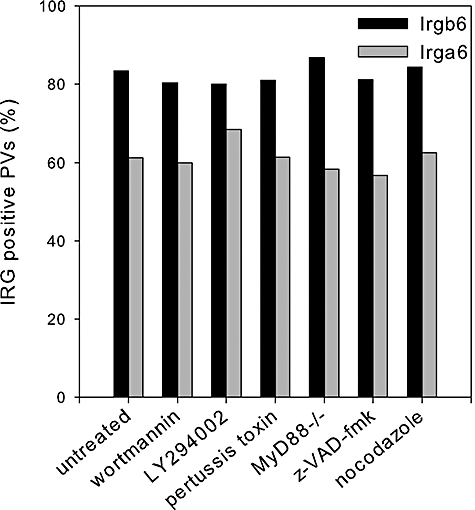
Vacuolar loading of IRG proteins is independent of major signalling systems and microtubules. C57BL/6 MEFs were induced with IFNγ and treated as described in *Experimental procedures* with inhibitors of PI3-kinase (wortmannin and LY294002), G protein-coupled receptors (pertussis toxin), caspases (z-VAD-fmk) and microtubule polymerization (nocodazole). Multiple TLR-mediated signals were excluded in IFNγ-induced MEFs from MyD88-deficient mice. The efficacy of each treatment was assayed as described in *Experimental procedures* and as shown in Fig. S2. Untreated, treated and MyD88-deficient cells were infected with *T. gondii* ME49 strain for 2 h and stained separately with antibody reagents against Irga6 (mAb 10D7) and Irgb6 (serum A20). The frequency of vacuoles detectably positive for Irga6 and Irgb6 was calculated as a percentage from 200–400 intracellular parasites. One representative experiment out of two independent repetitions is shown.

Transfer of Irga6 to the *T. gondii* PVM shortly after infection in IFNγ-induced cells is associated with GTP binding (Hunn *et al.*, 2008; Papic *et al.*, 2008) raising the possibility that activation of Irga6 results from the receipt of a cellular signal stimulated by infection. However disruption of major signalling systems failed to prevent the loading of IRG proteins onto the vacuoles of infecting *T. gondii*. We used wortmannin and Ly294002 to inhibit PI3 kinases, pertussis toxin to inhibit signal transmission from G protein-coupled receptors, MyD88-deficient MEFs to investigate a possible role of signalling through Toll-like receptor (TLR) signalling pathways and z-VAD-fmk to inhibit activation by caspases (see *Experimental procedures* for details). In no case was there any effect on the transfer of either Irga6 or Irgb6, analysed separately, to the PVM (Fig. 4; for controls see Fig. S2A and B). Irga6 has been reported to interact with Hook3, a Golgi-associated microtubule binding protein (Kaiser *et al.*, 2004) but dissociation of the microtubule network by nocodazole had no effect on the accumulation of IRG proteins at the PVM (Figs 4 and S2C). It therefore seems likely that transfer of IRG proteins to the vacuole is mediated by diffusion from the cytosolic pools of these proteins (Martens *et al.*, 2004; Martens and Howard, 2006) and activation of IRG proteins by GTP binding probably occurs at the PVM (Hunn *et al.*, 2008; Papic *et al.*, 2008).

### Vacuolar loading of the GKS subset of IRG proteins requires the autophagic regulator, Atg5

**Fig. 5:**
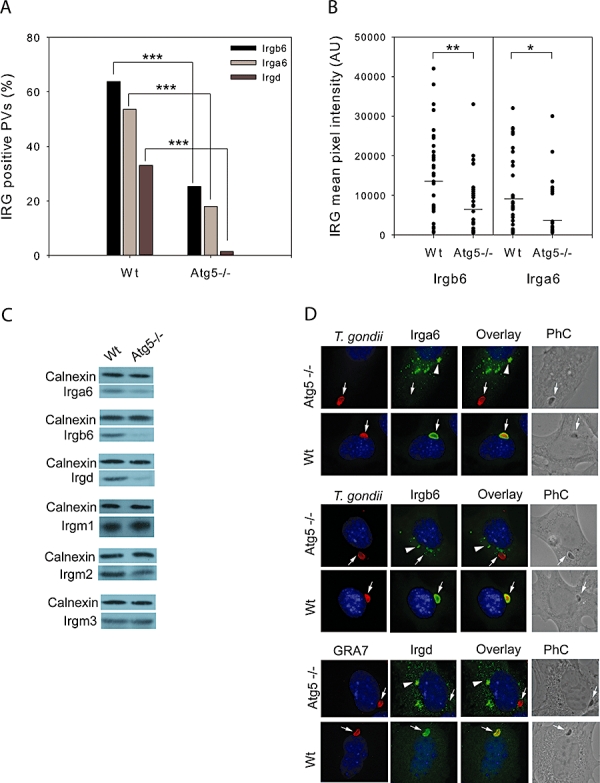
Atg5 influences loading of IRG proteins onto *T. gondii* PVs. A. IRG protein association with *T. gondii* ME49 PVs is reduced in Atg5^−/−^ fibroblasts. Wt and Atg5^−/−^ fibroblasts were induced for 24 h with IFNγ and infected with *T. gondii* ME49 strain for 2 h. Irga6-, Irgb6- and Irgd-positive vacuoles were detected by staining with mAb 10D7, serum A20 and serum 081/1 respectively. A total of 400–700 intracellular parasites were scored for each IRG protein in each cell line in 2–3 independent experiments. For statistical analysis the results for each condition were pooled. For all three IRG proteins the difference between wt and Atg5^−/−^ was highly significant by chi-squared test, *P* < 0.001, indicated by *** in the figure). B. The intensity of Irgb6 and Irga6 vacuolar loading is reduced in Atg5^−/−^ cells. Loading intensity was measured as described in *Experimental procedures* on at least 40 vacuoles from the experiment shown in (A). Horizontal bars represent the arithmetic mean values. By the Mann–Whitney test, for Irgb6 *P* < 0.01 for the difference between wt and Atg5^−/−^, indicated by ** in the figure, and for Irga6 *P* < 0.05, indicated by * in the figure). C. Irga6, Irgb6, Irgd and Irgm2 protein levels are reduced in Atg5^−/−^ MEFs while Irgm1 and Irgm3 are unaffected. Wt and Atg5^−/−^ fibroblasts were induced with IFNγ for 24 h and analysed by Western blot with antibody reagents detecting the following IRG proteins: Irga6 (mAb 10D7), Irgb6 (mAb B34), Irgd (serum 2078/3), Irgm2 (serum H53/3), Irgm1 (serum L115 BO) and Irgm3 (mAb anti-IGTP). D. IRG proteins form aggregates in IFNγ-induced Atg5^−/−^ MEFs. Cells were induced with IFNγ, infected with *T. gondii* ME49 strain and prepared for microscopical analysis as described in (A). Rabbit anti-*Toxoplasma* serum (upper and middle panels) or anti-GRA7 (lower panel) monoclonal antibody was used to identify the pathogen. Arrows indicate intracellular parasites. The arrowheads indicate the IRG protein aggregates. PhC, phase contrast.

IFNγ-induced mouse fibroblasts lacking the autophagic regulator, Atg5, restrict growth of avirulent ME49 strain of *T. gondii* weakly (Könen-Waisman and Howard, 2007), and Atg5-deficient macrophages stimulated with IFNγ and LPS are deficient in accumulating Irga6 at the PVM of the type II avirulent PTG strain of *T. gondii* (Zhao *et al.*, 2008). Figure 5A confirms for MEFs that significantly fewer ME49 vacuoles in the Atg5-deficient cells load with Irga6. In addition, the loading intensity of individual vacuoles with Irga6 was somewhat reduced (Fig. 5B). Loading of Irgb6 was greatly reduced in Atg5-deficient cells, and that of Irgd almost absent, showing that the lesion is not specific for Irga6 (Fig. 5A and B), but includes all three proteins of the GKS subfamily. The cellular levels of these IRG proteins were also reduced, especially so for Irgb6 and Irgd (Fig. 5C). Levels of the GMS subfamily proteins, Irgm1 and Irgm3, on the other hand were normal in Atg5-deficient fibroblasts. A slight reduction in Irgm2 may be attributed to the allele specificity of the antiserum used for its detection (see *Experimental procedures*). Like Zhao *et al.* (Zhao *et al.*, 2008), we noted that Irga6 was at least partially distributed in small aggregates in IFNγ-induced Atg5-deficient cells. These aggregates were stained with the monoclonal antibody, 10D7, specific for a conformation of Irga6 associated *in vivo* with the binding of GTP (Papic *et al.*, 2008) (Fig. 5D upper panels). Irgb6 and Irgd were also found in aggregates (Fig. 5D middle and lower panels). Proximity of Irga6 aggregates with LAMP1 in Atg5-deficient macrophages observed by Zhao *et al.* (Zhao *et al.*, 2008) was not seen in Atg5-deficient fibroblasts (Fig. S3).

### IRG proteins accumulate at the PVM in a consistent hierarchy

**Fig. 6:**
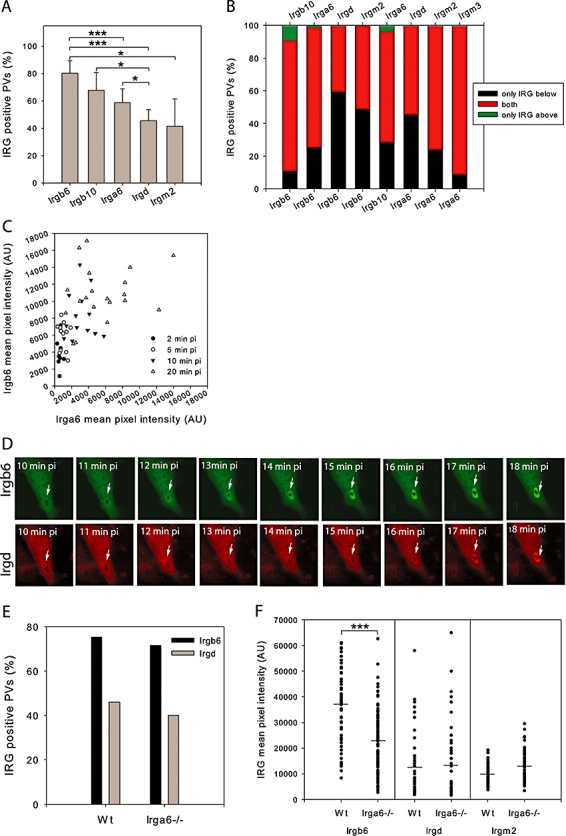
IRG proteins load in a consistent hierarchy on to the PV of *T. gondii* ME49 strain. A. Each IRG protein loads onto a characteristic proportion of vacuoles. Quantification of IRG-positive PVs (%) observed in IFNγ-induced 2 h *T. gondii* ME49 infected MEFs and gs3T3 cells assayed by immunocytochemistry using antibody reagents described in Fig. S4 and in *Experimental procedures*. At least three independent experiments were assayed and pooled and a minimum of 500 PVs counted for each IRG protein (error bars indicate the standard deviation between individual experiments). The statistical significances of the differences recorded were determined by Student's *t*-test and are shown on the figure (***P < 0.001; *P < 0.05). B. IRG proteins do not load at random onto each vacuole. IRG proteins loaded onto *T. gondii* PV were detected by co-staining with pairs of specific antibodies directed against IRG proteins at different positions in the hierarchy, using specific secondary reagents carrying different fluorochromes. Vacuoles loaded with one IRG protein were scored for possession of the second and vice versa. Vacuoles loaded with neither IRG protein were not included in the analysis. At least 100 positive vacuoles were counted for each pair of IRG proteins. Red bar segments give the percentage of vacuoles loaded with both IRG proteins in a given pair, while the green and black bar segments give respectively the percentages loaded with only the lower or only the higher member. The PV loading of pairs of IRG proteins is very strongly correlated such that nearly every vacuole loaded with an IRG protein lower down the hierarchy is also loaded with an IRG protein higher in the hierarchy. The full data are shown in Table S1. C. Irgb6 loads more heavily onto *T. gondii* vacuoles at early time points than Irga6. C57BL/6 MEFs were induced with IFNγ and infected with *T. gondii* ME49 strain. At indicated times after infection Irgb6 and Irga6 vacuole loading intensities were analysed simultaneously with specific primary antibodies (Irgb6, serum A20; Irga6, mAb 10D7) detected with secondary antibodies labelled with different fluorochromes. D. Irgb6 loads before Irgd on to the *T. gondii* ME49 strain PV. C57BL/6 MEFs were induced with IFNγ and transfected simultaneously with constructs expressing Irgb6-FLAG-EGFP and Irgd-ctag1-Cherry. After 24 h, cells were infected with *T. gondii* ME49 strain in microscope slide chambers and observed by live cell imaging for the accumulation of IRG proteins. Successive 1 min frames from one vacuole show Irgb6-FLAG-EGFP visibly loading several minutes before Irgd-ctag1-Cherry. E. Absence of Irga6 does not affect the proportion of vacuoles loaded with Irgb6 or Irgd. Irga6^−/−^ and wt MEFs were induced with IFNγ and infected with *T. gondii* strain ME49. 2 h after infection cells were stained with appropriate antibody reagents and the proportion of Irgb6 (mAb B34) and Irgd (serum 081/1) labelled vacuoles (out of 300 for each IRG protein in two independent experiments) was recorded. F. Intensity of PV loading by Irgb6 is significantly reduced in Irga6^−/−^ relative to wt MEFs (*P* < 0.001 by Mann–Whitney test, indicated by *** on the figure). IFNγ-induced Irga6^−/−^ and wt MEFs were infected with *T. gondii* ME49 strain. Two hours after infection slides were stained for Irgb6 (B34), Irgd (081/1) and Irgm2 (H53/3). At least 50 vacuoles loaded with each IRG protein were assayed for loading intensity from both cell types. The arithmetic means are given as horizontal lines.

Each IRG protein accumulates on a characteristic proportion of vacuoles. Thus Irga6 and Irgb6 both load onto the majority of vacuoles (Figs 1–5) (Martens *et al.*, 2005; Hunn *et al.*, 2008; Papic *et al.*, 2008), but Irgb6 invariably loads onto a higher proportion of vacuoles than Irga6 (Figs 4 and 5A). By discriminating two or three IRG proteins at a time on single intracellular parasites we examined the proportions of loaded vacuoles, as well as the coupled or uncoupled distribution, of Irga6, Irgb6, Irgb10, Irgd, Irgm2 and Irgm3. All antibody reagents used showed saturation binding to their vacuolar targets (Fig. S4 and A. Khaminets and S. Könen-Waisman, unpublished data). Four different immunoreagents failed to find any vacuolar loading of Irgm1, despite its strong association with resistance to *T. gondii* (S. Könen-Waisman, unpublished data and see also Martens *et al.*, 2005). Vacuolar loading by five IRG proteins fell into a consistent hierarchy in the order Irgb6 > Irgb10 > Irga6 > Irgm2 ≈ Irgd (Fig. 6A). Irgm3 frequency was low, probably below Irgd/Irgm2, but a value is not shown because the loading intensity was low and difficult to resolve from the surrounding ER. The hierarchy was reproducible except for the relative positions of Irgd and Irgm2 and was independent of the reagents used.

If IRG proteins load randomly onto vacuoles, then essentially every vacuole should be loaded with one or the other and most with more than one in random combinations. However when the loading of two or more members of the family was scored on each vacuole it was clear that they were strongly correlated. In Fig. 6B this is illustrated for pairs of IRG proteins at different positions in the loading hierarchy. Invariably, vacuoles loaded with the IRG protein lower in the hierarchy are very largely or completely included in the set of vacuoles loaded with the IRG protein higher in the hierarchy (The full data from which Fig. 6B was generated are given in Table S1). Thus 98.4% (612/622) of vacuoles loaded with Irga6 were also loaded with Irgb6, while 100% (390/390) of vacuoles loaded with Irgd were also loaded with Irga6 (Fig. 6B and Table S1). The two highest members of the hierarchy, Irgb6 and Irgb10 were about 90% correlated. The effect of the correlated loading of IRG proteins is that a significant number of vacuoles (normally 10–20%) did not load with any IRG proteins at all while about 40% of the vacuoles (i.e. those loaded with Irgd or Irgm2) were loaded with all the IRG proteins studied.

The correlated loading behaviour suggested that IRG proteins might assemble on the vacuoles in sequence. Analysis of Irga6 and Irgb6 accumulation on to ME49 *T. gondii* strain vacuoles in double-labelled samples taken shortly after infection showed that the earliest increase in label is due to accumulation of Irgb6, followed several minutes later by Irga6 (Fig. 6C). Live cell observation confirmed this result for Irgd, which loaded later than Irgb6 or Irga6. ME49 strain vacuoles were observed in IFNγ-induced MEFs transfected with Irgd-ctag1-Cherry together with either Irgb6-FLAG-EGFP (Fig. 6D) or Irga6-ctag1-EGFP (Fig. S5). Invariably the IRG protein higher in the hierarchy was the first to load, followed after a variable delay of several minutes by the lower member (Fig. 6D, Irgb6 followed by Irgd; Fig. S5, Irga6 followed by Irgd). In no case were vacuoles found to load with the lower member first.

In Irga6-deficient MEFs (Martens *et al.*, 2005) the loading of Irgd to Irgb6-positive vacuoles was not affected. The hierarchical series formed as usual, but without Irga6 (Fig. 6E). The inclusion relationships described above were also preserved in Irga6-deficient cells (S. Könen-Waisman, unpublished data). There was, however, a numerically highly significant tendency (*P* < 0.0001 by Mann–Whitney test) towards reduced Irgb6 vacuole loading intensity in Irga6-deficient cells relative to the wild-type (wt) cells (Fig. 6F), suggesting that the presence of Irga6 may stabilize the loading of Irgb6. No effect on that scale was seen for Irgd or Irgm2 (Fig. 6F).

### Cooperative interactions in the loading of IRG proteins onto the *T. gondii* PVM

**Fig. 7:**
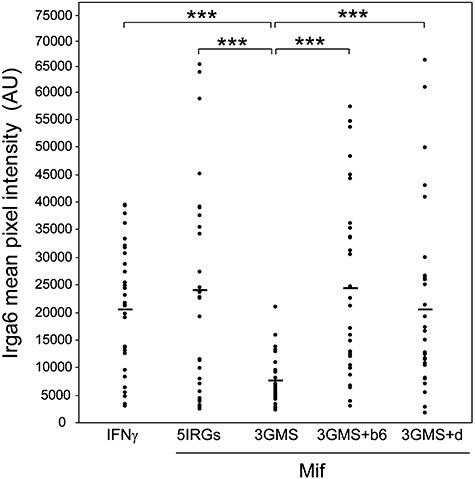
Loading of Irga6 at the *T. gondii* ME49 strain PV is enhanced by the presence of other IRG proteins of the GKS group. gs3T3-Irga6 cells were induced with IFNγ or Mifepristone. At the same time, Mifepristone-induced cells were transfected with pools of constructs (see *Experimental procedures* for experimental details) expressing either the three GMS proteins, (Irgm1, Irgm2 and Irgm3) alone to permit access of Irga6 to the PV (3GMS) or, in addition to the 3GMS proteins, also Irgb6 (3GMS + b6), Irgd (3GMS + d) or both Irgb6 and Irgd (5IRGs). After 24 h, cells were infected with *T. gondii* ME49 strain for 2 h. Irga6 was detected at the PV in transfected cells using mAb 10E7 in immunofluorescence. Transfected cells were identified by staining for Irgm2 with the H53/3 serum. The arithmetic means are given as horizontal lines. Vacuolar loading of Irga6 was significantly enhanced by addition of Irgb6 or Irgd. The *P*-values are given on the figure (***P < 0.001).

The three GMS proteins, Irgm1, Irgm2 and Irgm3, characterized by a unique substitution of methionine for lysine in the G1 motif of the nucleotide-binding site (Boehm *et al.*, 1998), control the GTPase cycle of the conventional GKS proteins, Irga6, Irgb6 and Irgd (Hunn *et al.*, 2008). In the absence of the GMS proteins, Irga6 and Irgb6 form nucleotide-dependent cytoplasmic aggregates that are probably caused by premature GTP binding and activation (Hunn *et al.*, 2008; Papic *et al.*, 2008). Irga6, expressed alone in 3T3 cells from a Mifepristone-inducible construct, was unable to relocate to the PVM of infecting *T. gondii* strain, but vacuolar location was restored if the three GMS proteins were also introduced by transfection (Hunn *et al.*, 2008). However the frequency of Irga6-loaded vacuoles was low (Hunn *et al.*, 2008) and accumulation of Irga6 at the PVM was weak (Fig. 7 and J.P. Hunn, unpublished data). The co-ordinated loading described above hinted that Irgb6 (and/or other GKS proteins) might be necessary for complete loading of Irga6. We therefore reconstituted Mifepristone-induced gs3T3-Irga6 fibroblasts with constructs expressing Irgb6, Irgd or both as well as the three GMS proteins and determined the Irga6 signal intensity on *T. gondii* PVM 2 h after infection. Coexpression of either Irgb6 or Irgd or both caused a significant increase in the Irga6 signal at the PVM without increasing the frequency of Irga6-loaded vacuoles (Fig. 7, Hunn *et al.*, 2008 and J.P. Hunn, unpublished data). Thus other IRG proteins of the GKS group are required for efficient Irga6 loading of the PVM. Unlike Irga6, Irgd does not load at all on to the PVM if only the three GMS proteins are also present, but loads if Irgb6 is also present (J.P. Hunn, unpublished data). Thus the loading of the PVM by GKS proteins is a highly cooperative process, with Irgb6 and probably also Irgb10 arriving as pioneers and subsequently becoming stabilized by the arrival of Irga6 and Irgd. In this context, it may be significant that both Irgb6 and Irgb10 can load, albeit inefficiently, onto *T. gondii* vacuoles in the absence of other IRG proteins (Hunn *et al.*, 2008, Fig. 7 and J.P. Hunn, unpublished data). A different explanation for the presence of Irgm2 and Irgm3 at the vacuole is presented in the *Discussion*.

### Reduced loading of IRG proteins on to the PVM of virulent strains

**Fig. 8:**
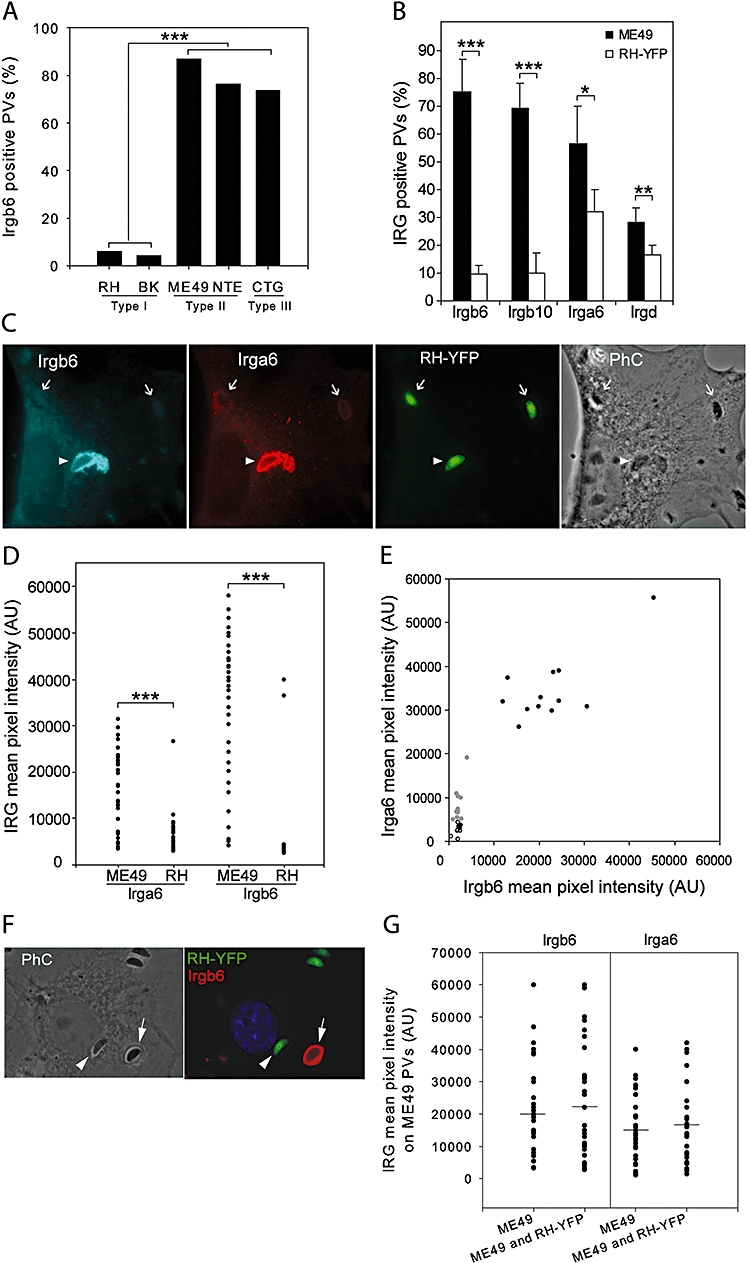
Accumulation of IRG proteins on the PVM is reduced in virulent *T. gondii* infection. A. IFNγ-induced MEFs were infected for 2 h with type I virulent (RH and BK), type II (ME49 and NTE) and type III (CTG) avirulent *T. gondii* strains and assayed microscopically for Irgb6-positive vacuoles (serum A20). Irgb6-positive PVs were counted for each parasite strain from 400–600 intracellular parasites in two independent experiments and pooled. The differences between the avirulent types II and III strains and the virulent type I strains are highly significant by chi-squared test (***P < 0.001). B. gs3T3 cells were induced with IFNγ and infected with *T. gondii* ME49 strain (black bars) or RH-YFP strain (white bars). Numbers of Irgb6-, Irgb10-, Irga6- and Irgd-positive PVs were counted in 3–6 experiments for each IRG protein and *T. gondii* strain and given as a percentage of intracellular parasites. More than 200 intracellular parasites were counted blind per experiment. The mean percentages of positive vacuoles of ME49 or RH-YFP type for each IRG protein are shown. Error bars indicate the standard deviations. The significances of the differences between loading of ME49 and RH-YFP vacuoles are given on the figure (***P < 0.001, **P < 0.01, *P < 0.05, by Student's *t*-test). C. C57BL/6 MEFs were induced with IFNγ and infected with RH-YFP. Irgb6 (blue) and Irga6 (red) were detected in immunofluorescence with serum A20 and mAb 10E7 respectively. Intracellular fluorescent parasites (RH-YFP, green) identified in phase contrast (PhC) are indicated by white arrowheads (strongly IRG-positive) and arrows (weakly IRG-positive). D. gs3T3 fibroblasts were induced with IFNγ and infected with either ME49 or RH-YFP *T. gondii* strains. Mean fluorescence intensities of Irga6 (serum 165/3) and Irgb6 (serum A20) signals at the PVM were quantified as described in Fig. S1 and *Experimental procedures*. Thirty-five random PVs per data set were quantified blind. For both Irga6 and Irgb6, the different loading intensities on ME49 and RH-YFP vacuoles were highly significant (***P < 0.001). E. C57BL/6 MEFs were induced with IFNγ and infected with *T. gondii* RH-YFP strain. Mean fluorescence intensities of Irga6 and Irgb6 were measured for selected PVs expressing no detectable (open circles), weak (grey filled circles) or strong Irga6 staining (black filled circles). The fluorescent intensity profiles of five representative PVs per group are displayed in Fig. S6. F. Photomicrograph of an IFNγ-stimulated MEF shown 2 h after double infection with ME49 strain (indicated by arrowhead) and RH-YFP strain *T. gondii* (green, indicated by arrow). The ME49 strain parasite shows intense Irgb6 (serum A20, red) accumulation at the PV while the RH-YFP in the same cell has no Irgb6 on the PV. G. IFNγ-stimulated MEFs were infected with *T. gondii* ME49 strain alone or simultaneously with ME49 and RH-YFP strains. Irgb6 (detected by serum A20) and Irga6 (detected by mAb 10D7) fluorescence intensities were measured on at least 30 ME49 PVs in singly and doubly infected cells. ME49 and RH-YFP were discriminated by the fluorescent signal from RH-YFP. The arithmetic means are given as horizontal lines. The loading of Irga6 and Irgb6 onto PVs of avirulent ME49 strain *T. gondii* was unaffected by the simultaneous presence of virulent RH-YFP.

Reduced loading of Irgb6 on virulent type I strain RH PVs has recently been reported in MEFs and macrophages, correlating with reduced vacuolar disruption (Zhao *et al.*, 2009a,b,c;). The data shown above were all based on infection of cells with the avirulent *T. gondii* strain, ME49. As shown in Fig. 8A, two type I strains, BK and RH, gave grossly defective loading of Irgb6, while two avirulent type II strains, ME49 and NTE, and an avirulent type III strain, CTG, showed the familiar high frequency of Irgb6-loaded vacuoles. Loading of all IRG proteins was attenuated on RH strain vacuoles (Fig. 8B) but two different effects were apparent. Irgb6 and Irgb10 loaded onto very few vacuoles, while for Irga6 or Irgd the number of loaded vacuoles was significantly but not dramatically reduced but the amount loaded per vacuole was generally much lower, documented for Irga6 in Figs 8C and D and S6. The few vacuoles loaded with Irgb6 were loaded very heavily (Fig. 8C and D) and were additionally all intensely loaded for Irga6 (Figs 8E and S6) and Irgd (J.P. Hunn and S. Könen-Waisman, unpublished data). In addition to their implications for the nature of virulence, these results also support the concept that Irgb6 and Irgb10 function as loading pioneers. In their absence, the remaining IRG proteins have difficulty gaining a foothold on the vacuole. In IFN-induced MEFs co-infected with RH-YFP and unlabelled ME49, ME49-containing vacuoles could be intensely coated with Irgb6 while RH-containing vacuoles in the same cell had none (Fig. 8F), and the loading intensity of Irgb6 and Irga6 on ME49 PVs was unaffected by the presence of RH-YFP vacuoles (Fig. 8G). It is therefore unlikely that a diffusible molecule from the virulent strain is responsible for reduced Irgb6 loading. Our results and conclusions confirm those of Zhao *et al.* (Zhao *et al.*, 2009c).

**Fig. 9:**
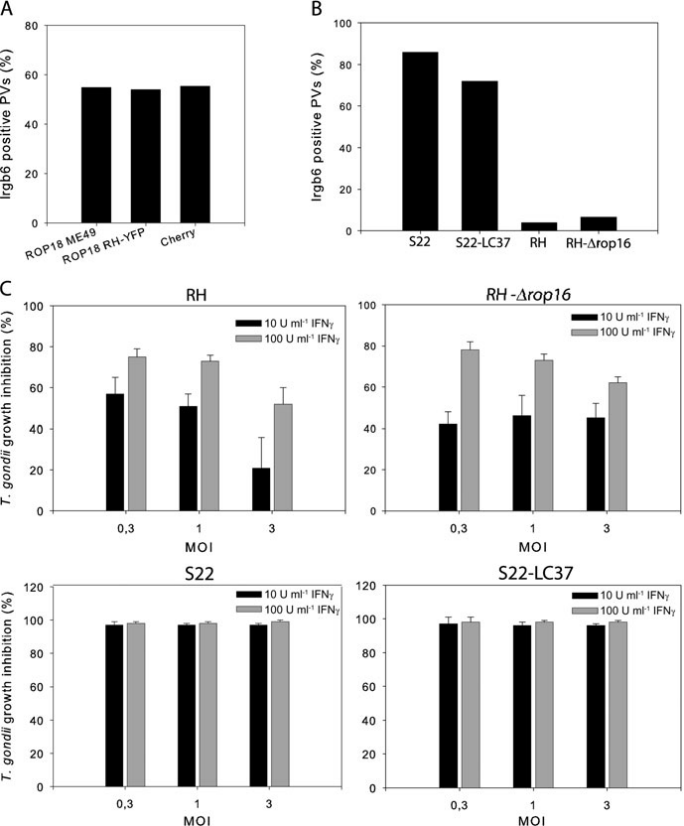
ROP18, ROP16 and ROP5 virulence-associated *T. gondii* proteins do not affect IRG-mediated control of the parasite. A. Ectopically expressed ROP18 does not affect loading of *T. gondii* ME49 PVs with Irgb6 in infected L929 cells. L929 cells were induced with IFNγ, transfected with pGW1H expression plasmids encoding the mature form of ROP18 from either ME49 or RH-YFP *T. gondii* strains, or pmCherry-N3 as a transfection control and infected for 2 h with *T. gondii* ME49 strain. Cells were stained for Irgb6 (serum A20) and for Ty-tag to identify the transfected cells. Irgb6-positive vacuoles in ROP18-Ty-tag and Cherry-positive cells were enumerated. A total of approximately 700 vacuoles were scored in two independent experiments. B. ROP16 and ROP5A–D do not affect Irgb6 loading onto *T. gondii* PV. IFNγ-stimulated MEFs were infected with the S22-LC37 *T. gondii* strain expressing four ROP5 and two other genes (see also *Experimental procedures* and main text), RH-Δrop16 and control parental strains S22 and RH for 2 h. Irgb6-positive PVs (stained with serum 141/1) were quantified from 350–500 intracellular parasites. The results shown are pooled from two independent experiments. C. The IFNγ-mediated growth inhibition of RH-Δrop16 and S22-LC37 *T. gondii* strains are comparable in each case to the inhibition of the respective RH and S22 control strains in MEFs. Proliferation of *T. gondii* strains was measured by ^3^H-uracil incorporation and presented as a percentage of *T. gondii* growth inhibition, as described in *Experimental procedures*. Black and grey bars represent the extent of parasite growth inhibition at 10 and 100 U ml^−1^ of IFNγ cell stimulation respectively. See Fig. S7 for untransformed data.

Polymorphic *T. gondii* genes encoding rhoptry kinases and pseudokinases of the ROP2 family secreted into the host cell at the point of entry (Carruthers and Sibley, 1997) contribute to the differential virulence phenotypes of *T. gondii* types I, II and III strains (Saeij *et al.*, 2006; Taylor *et al.*, 2006; El Hajj *et al.*, 2007a). ROP18 accumulates on the PVM of the invading parasite and ROP18 expressed in infected cells by transfection also targets the PVM (El Hajj *et al.*, 2007a). ROP18 differs in amino acid sequence between all three virulence types (Saeij *et al.*, 2006) and is under-expressed in avirulent type III parasites. However expression by transfection of mature form ROP18 from the virulent RH strain into IFNγ-induced L929 fibroblasts had no effect on the number of ME49 vacuoles loaded with Irgb6 compared with cells expressing either ME49-derived ROP18, or Cherry as a transfection control (Fig. 9A).

Two further polymorphic ROP2 family members have been implicated in virulence-related behaviour. ROP16 alleles differentially affect cellular signal transduction pathways responsible for inflammation *in vivo* (Saeij *et al.*, 2007) and have some impact on virulence (Saeij *et al.*, 2006), while a virulence QTL on Chromosome XII embraces the ROP5 locus (Saeij *et al.*, 2006). This locus, originally annotated as a single gene called ROP5 (TGME49_108080), contains five nearly identical ROP2 family members dubbed ROP5A–E (http://www.toxodb.org and J.C. Boothroyd, unpublished data; Saeij *et al.*, 2006). We assayed the loading of Irgb6 onto the PVMs of virulent RH strain deficient in ROP16 (RH-Δrop16) compared with the RH wt control, and onto the PVMs of an avirulent strain, S22, transgenic for cosmid LC37 (S22-LC37) carrying a segment of the RH genome encompassing ROP5A–D as well as two other predicted gene sequences (TGME49_108070 and TGME49_108060). Neither genetically modified parasite was affected in its behaviour *vis-à-vis* the IRG resistance mechanism (Fig. 9B). RH-Δrop16 showed the same deficit in Irgb6 loading as the wt virulent RH strain. Likewise, *T. gondii* S22-LC37 behaved no differently from the avirulent parental S22 strain (Fig. 9B and S. Könen-Waisman, unpublished data). RH-Δrop16 replication was no better controlled by IFNγ treatment of the host MEFs than RH itself, while S22-LC37 was as well controlled by IFNγ as parental S22 (Figs 9C and S7).

## Discussion

The PVM of *T. gondii* parasites infecting IFNγ-induced mouse cells becomes coated with multiple members of the IRG family of IFN-inducible resistance GTPases. The process is important for resistance because IRG proteins are able to disrupt the PVM (Martens *et al.*, 2005), and vacuolar disruption leads to the rapid death of the enclosed parasite (Zhao *et al.*, 2009a). The IFNγ-dependent resistance mechanism largely fails when the infecting *T. gondii* is a virulent type I parasite (Zhao *et al.*, 2009a,b,c;). We show here that this is because IRG proteins fail to load effectively on to the PVM. To understand the mechanistic basis for *T. gondii* virulence and avirulence in mice, we have therefore begun to dissect the process by which IRG proteins load on to the PVM.

### The initiation of loading

The earliest infecting parasites begin to accumulate IRG proteins on the PVM within 2.5 min of addition to culture, but the proportion of IRG-loaded vacuoles rises for about 1 h (Fig. 1A). There is a delay of highly variable length before IRG proteins begin to accumulate (Fig. 2) but once initiated, IRG protein accumulation is roughly linear for up to 1 h (Fig. 2C). Cytoplasmic Irga6 molecules areprobably GDP-bound while those that accumulate on the PVM are GTP-bound (Hunn *et al.*, 2008; Papic *et al.*, 2008). Extensive GTP-dependent interactions between IRG protein molecules, documented elsewhere (Hunn *et al.*, 2008) and in the present study, would allow for the formation of homo- and hetero-oligomers at the PVM. The variable delay in initiating loading suggests that the rate-limiting step for colonization of the PVM is the initiation of oligomerization by GTP binding at the PVM. Once oligomerization begins, further molecules of GTP-bound IRG proteins can be added with high efficiency.

Our experiments indicate that the transfer of IRG proteins to the PVM is not triggered, for example by phosphorylation, via the main cell-signalling systems activated by infection (Figs 4 and S2). No effect was seen following inhibition of classical PI3 kinases, of G protein-coupled receptors, of caspases, or by removal of the MyD88 adaptor of TLR signalling even though the TLR system is stimulated by several independent *Toxoplasma* elicitors (reviewed in Egan *et al.*, 2009). We have, however, not yet explored the possibility of a trigger emanating from a TLR via a MyD88-independent pathway or from a cytoplasmic detector such as one of the NALP proteins.

The microtubule cytoskeleton is not directly involved in transporting IRG proteins to the PVM, despite evidence that Irga6 interacts with the microtubule binding protein, Hook3 (Kaiser *et al.*, 2004) as nocodazole treatment failed to block vacuolar loading by Irga6 or Irgb6 (Fig. 4 and Fig. S2). Thus the infection process probably does not trigger the activation or active transport of IRG proteins via a host mechanism. We suggested recently that the characteristics of the *Toxoplasma* vacuolar membrane directly stimulate the re-localization of IRG proteins (Hunn *et al.*, 2008). The key issue is: why do IRG proteins concentrate on this membrane? All the GKS proteins, including the pioneer, Irgb6, could initially reach the PVM by simple diffusion as they have large cytosolic pools (Martens *et al.*, 2004). It is not yet known how Irgb6, lacking evident membrane attachment signals, associates with PV membranes. Perhaps an early interaction with Irgb10, which has a perfect myristoylation motif (Bekpen *et al.*, 2005), assists Irgb6 targeting. Myristoylation of Irga6 is essential for its own vacuolar targeting (N. Papic, unpublished data). However to avoid premature activation on endogenous cellular membranes the GKS proteins require the presence of all three of the membrane-bound GMS proteins (Hunn *et al.*, 2008), which are distributed differentially on Golgi, ER (Taylor *et al.*, 1997; Martens *et al.*, 2004; Martens and Howard, 2006) and lysosomal membranes (Zhao *et al.*, 2010). The initial absence of GMS proteins on the PVM immediately after infection may therefore provide a window for spontaneous activation at this membrane.

### Variation of loading intensity and the loading hierarchy

There is remarkable variation in loading intensity even within single cells for both Irga6 and Irgb6 (Figs 1C and 3A and B), with some vacuoles apparently failing completely to acquire any IRG proteins over many hours (Fig. 2C, Video S2 and Y.O. Zhao, unpublished data). Vacuoles may become progressively more resistant to IRG loading with time after infection, perhaps through the maturation of a parasite-determined defence mechanism. Such an effectcould be related to the mechanism of virulence discussed below.

Our data (Fig. 6A and B and Table S1) establish that different IRG proteins assemble cooperatively on the PVM in a distinct order, with Irgb6 and Irgb10 as pioneers. If vacuoles become resistant to IRG loading with time after infection, then this process probably affects the loading of Irgb6 and Irgb10. The strong cooperativity of loading would thereby ensure that a proportion of vacuoles would remain free of IRG proteins. We have shown recently that all three GMS proteins, Irgm1, Irgm2 and Irgm3, are required for loading of Irga6 onto the PVM of ME49 strain *T. gondii* (Hunn *et al.*, 2008). However this loading is inefficient unless other GKS proteins, Irgb6 and/or Irgd, are also present (Fig. 7). The present study confirms this, as nearly all Irga6-loaded vacuoles were also positive for Irgb6 and Irgb10 (Fig. 6B and Table S1). Finally, Irga6 loading of Irgb6-negative vacuoles containing the virulent RH strain is far less efficient than the loading of the rare Irgb6-positive vacuoles (Fig. 8). These results suggest that Irga6 accumulation is directly facilitated or stabilized by the presence of Irgb6 and Irgd, and, we anticipate, of Irgb10 as well. This effect may well be due to the formation of mixed GTP-dependent oligomers because at least Irga6 and Irgb6 interact strongly in a nucleotide-dependent manner in yeast 2-hybrid assays (Hunn *et al.*, 2008). That stabilizing interactions are reciprocal is suggested by the highly significant reduction in Irgb6 signal at the PV (*P* < 0.0001) in the absence of Irga6 (Fig. 6F). We conclude that Irgb6 and probably also Irgb10 are ‘pioneers’ in being able to load at least to some extent by themselves. Their loading is then reinforced by the arrival of the other GKS proteins. Because Irgd loading is not detectably affected by loss of Irga6, Irgd either normally does not interact with Irga6 on the vacuole or alternatively in the absence of Irga6 it interacts with other members of the hierarchy. There is at present no reason to favour either of these alternatives over the other.

Whether such thinking is appropriate for the association of the regulatory GMS proteins with the PVM is not clear. The GMS proteins have low (Irgm2, Irgm3) or absent (Irgm1) free cytosolic pools (Martens *et al.*, 2004; Martens and Howard, 2006) so access to the vacuole by free diffusion is limited. Irgm3 is associated with the ER membrane, and Irgm3 association with the PVM could be due to the investment of the PV by ER cisternae (Melzer *et al.*, 2008) or by direct fusion between PV and ER membranes (Goldszmid *et al.*, 2009). Irgm2 is exclusively associated with Golgi membranes (Martens and Howard, 2006), but a secondary association with the PVM via Golgi-ER exchange is possible. However Irgm1, with no cytosolic pool (Martens *et al.*, 2004), is also associated prominently with the Golgi (Martens *et al.*, 2004; Butcher *et al.*, 2005), but is never detected on the PVM. The GMS proteins are essential regulators of the GKS subfamily, maintaining these latter in the GDP-bound state before infection probably by direct GDP-dependent interaction (Hunn *et al.*, 2008). After infection, the PVM is a site of highly concentrated GKS proteins, initially in the GTP-bound state, but presumably exerting their function at the vacuole via GTP hydrolysis. Thus the Irgm2 and Irgm3 proteins found at the vacuole may reflect binding of the small cytosolic pools of these proteins to PVM-localized GKS molecules that have hydrolysed their GTP to GDP but have not dissociated from the vacuole. This model is consistent with the absence of Irgm1 from the PVM and with the dependence of PV binding of both Irgm2 and Irgm3 on the presence of GKS proteins (Hunn *et al.*, 2008).

C57BL/6 mice possess other GKS proteins that we have not yet analysed in detail (Bekpen *et al.*, 2005). We have, however, indications that some of these also associate with avirulent *T. gondii* vacuoles (J.C. Howard and R. Lange, unpublished data). How these will fit into the loading hierarchy remains to be seen.

In view of IRG function in *T. gondii* resistance it is of great interest whether intense loading of PVs with multiple IRG proteins favours PVM vesiculation and parasite death. Although critical experiments are still missing, the correlation between reduced PV loading with IRGs and reduced vacuole disruption in virulent *T. gondii* infection indicates that this might indeed be the case. Furthermore, control of *T. gondii* is somewhat reduced in Irga6-deficient cells (Martens *et al.*, 2005) and *in vivo* in Irgd-deficient mice (Collazo *et al.*, 2001). Thus the significance of multiple IRG protein loading seems to be reflected in the non-redundancy of the two GKS proteins that have been knocked out so far.

### The autophagic regulator, Atg5

IFNγ-induced mouse fibroblasts lacking the autophagic regulatory protein, Atg5, do not control avirulent *T. gondii* replication efficiently (Könen-Waisman and Howard, 2007) and Irga6 fails to load the PV in Atg5-deficient macrophages, forming cytosolic aggregates (Zhao *et al.*, 2008). Thus Atg5 may have a specific function in loading Irga6 onto the PV. However, we find in Atg5-deficient fibroblasts that defect in vacuolar loading involves all the tested GKS proteins (Fig. 5). Not only Irga6 but also Irgb6 and Irgd formed aggregates in the Atg5-deficient IFNγ-induced fibroblasts, and the Irga6 aggregates bind the GTP-dependent monoclonal antibody 10D7 (Fig. 5) (Papic *et al.*, 2008). GTP binding by the GKS subfamily of IRG proteins is normally controlled by dynamic interactions with the GMS subfamily (Hunn *et al.*, 2008), and when such control is lost, Irga6 and Irgb6 form GTP-dependent aggregates in the cytosol and do not localize to the PVM (Hunn *et al.*, 2008; Henry *et al.*, 2009a). Furthermore, the reduction in GKS protein levels in Atg5-deficient cells (Fig. 5) recalls the reduced GKS protein levels found in IFNγ-induced cells from Irgm1- and Irgm3-deficient mice (Henry *et al.*, 2009a). Taken together we conclude that Atg5 has an effect on IRG protein loading onto *T. gondii* PV, but that this is indirect as absence of Atg5 causes inappropriate activation of the GKS proteins resulting in their mislocalization and aggregation and presumably subsequent degradation. Our findings suggest that Atg5 expression may be necessary for the normal function of the three GMS proteins in regulating nucleotide exchange in the GKS subfamily on the membranes of cytoplasmic compartments (Hunn *et al.*, 2008). Which if any of the increasing range of activities attributed to Atg5 (Hanada *et al.*, 2007; Geng and Klionsky, 2008) could be responsible for this activity is unclear.

### The association of IRG proteins with the PVM from virulent *T. gondii* strains

Reduced loading of IRG proteins onto the vacuoles of virulent strains was recently reported by Zhao *et al.* (Zhao *et al.*, 2009c) in activated mouse peritoneal macrophages. They did not report the minority of vacuoles with heavy IRG loading seen here in fibroblasts (Fig. 8C–E), perhaps reflecting the two rather different cellular systems. The failure of IRG loading on the great majority of vacuoles derived from virulent *T. gondii* strains (Fig. 8) suggests specific inhibition by the parasite. Irgb6 and Irgb10 are most affected, the two proteins that load earliest and most efficiently on vacuoles of an avirulent strain (Fig. 6). Targeting of Irgb6 and Irgb10 would be an economical defence strategy against IRG proteins, as in their absence vacuolar loading by other IRG proteins becomes inefficient. The inhibition of Irgb6 loading by the virulent strain, although striking, is not complete. Approximately 10% of vacuoles load heavily with Irgb6 and Irga6 and behave like the IRG-loaded vacuoles of an avirulent strain (Zhao *et al.*, 2009a): they disrupt, the parasite dies and the infected cell subsequently dies by necrosis (Y.O. Zhao, unpublished data). We proposed above that the failure of a proportion of vacuoles to load with IRG proteins even during infection with avirulent *T. gondii* could be due to some form of maturation towards resistance to IRG loading induced by the parasite. An exaggerated or accelerated form of the same process could account for the situation with virulent strains.

The fact that inhibition is not dominant in mixed infections with virulent and avirulent strains (Fig. 8F and G) suggests that the inhibitory effect is mediated directly at the vacuolar membrane. Our experiments argue against a role for the rhoptry proteins, ROP5, ROP16 and ROP18 in this effect, although all three have been implicated genetically in virulence differences between *T. gondii* strains. For ROP18, our results (Fig. 9) confirm for fibroblasts what was reported recently by Zhao *et al.* for macrophages (Zhao *et al.*, 2009c). ROP18 seemed initially a good candidate because it associates with the PVM and is an active kinase. However both sequence and expression results group types I and II strains alleles together with type III alleles as the outlier, while IRG-related virulence effects group the avirulent types II and III strains together with type I as the outlier. Unlike ROP18, ROP16 is not localized at the PVM, has a known action on host inflammatory kinases and is associated with only modest mouse mortality in genetic tests (Saeij *et al.*, 2006; 2007;). Additionally, the ROP16 alleles of types I and III strains confer an indistinguishable phenotype in STAT activation assays and are nearly identical at the protein level (Saeij *et al.*, 2007). Like ROP18, the ROP5 genes encode ROP2 family members associated with the PVM (El Hajj *et al.*, 2007b) but none of the five ROP5 genes appears to encode a catalytically active kinase (El Hajj *et al.*, 2006 and J.C. Boothroyd, unpublished data). By the IRG-related assays described here S22-LC37, carrying four of the five ROP5 alleles of the RH strain, behaves like the avirulent parental S22 strain. In addition, the types I and III alleles for the entire ROP5A–E cluster are highly similar while type II and III, which are similar in IRG recruitment, carry very different versions of this cluster (toxodb.org and J.C. Boothroyd, unpublished data). Thus none of the tested ROP proteins are strong candidates a priori for the virulence-associated IRG loading effects. Further genetic studies will surely reveal the *T. gondii* genes involved.

IRG proteins are major players in *T. gondii* resistance in mice and show a high degree of cooperative activity (Hunn *et al.*, 2008 and this paper), consistent with their non-redundancy in single gene knockouts. Because *T. gondii* virulence is also determined by epistatic interactions between alleles at multiple loci (Saeij *et al.*, 2006) the single-locus gain-and-loss cases analysed here may fail to reproduce the complexity of the natural situation.

*Toxoplasma gondii* is not the only organism where virulence differences are associated with the mouse IRG system. The human pathogen, *Chlamydia trachomatis,* is resisted by interferon-induced mouse cells through the action of at least three IRG proteins, Irgm1, Irgm3 and Irgb10 (Bernstein-Hanley *et al.*, 2006; Miyairi *et al.*, 2007; Coers *et al.*, 2008) while the mouse-specific race, *Chlamydia muridarum* is not controlled. Control of *C. trachomatis* and lack of control of *C. muridarum* is correlated with the presence or absence of IRG proteins, specifically Irgb10, on the *Chlamydia* inclusion (Coers *et al.*, 2008). *C. muridarum* and *C. trachomatis* are over 90% identical at the nucleotide level and share over 99% of their open reading frames (Stephens *et al.*, 1998; Read *et al.*, 2000), while virulent type I and avirulent types II and III *T. gondii* clonal lineages are greater than 95% identical at the nucleotide level (Boyle *et al.*, 2006), thus facilitating in both cases the search for loci responsible for these large differences in virulence. Probably when the resistance mechanisms of the virulent strains of both parasites are revealed they will contribute to the elucidation of the IRG resistance mechanism itself.

### Is Irgm1 a special case?

The loading of multiple IRG proteins onto vacuolar inclusions has been documented for only two intracellular organisms, *T. gondii* and *C. trachomatis.* Mice deficient in several individual IRG proteins show susceptibility phenotypes to these pathogens. However, IRG-dependent resistance in mice has been claimed for many other protozoal and bacterial pathogens based always and only on mice deficient in Irgm1, the GMS subfamily regulator protein that does not localize to the *T. gondii* vacuole (Martens *et al.*, 2005; Taylor, 2007). Because Irgm1 has been shown to relocate to phagocytic cups and phagosomes during particle uptake (MacMicking *et al.*, 2003; Martens *et al.*, 2004) it has been proposed that this IRG protein can implement a direct cell-autonomous attack on phagocytosed pathogens (MacMicking *et al.*, 2003). However, Irgm1 deficiency is associated with haemopoietic stem cell failure (Feng *et al.*, 2008), defective proliferative potential of T lymphocytes in animals infected with highly immunostimulatory parasites such as *Mycobacteria* (Feng *et al.*, 2004; Santiago *et al.*, 2005) and with cell-autonomous anomalies of macrophage behaviour (Henry *et al.*, 2007; 2009b;). Thus susceptibility of Irgm1-deficient mice to multiple organisms may follow from the immunodeficiency rather than from loss of a direct action by Irgm1 against pathogen inclusions of various kinds. Indeed, the complex deficiency of the Irgm1-deficient mouse is completely reversed in the Irgm1/Irgm3 double-deficient mouse (Henry *et al.*, 2009a), showing that neither the presence of Irgm1 nor of Irgm3 is in fact required for resistance to the ‘Irgm1-dependent’ organisms such as *Salmonella* or *Mycobacteria*. The evidence now suggests that the generalized, IFNγ-dependent immunodeficiency caused by loss of Irgm1 is due to loss of one of the three essential regulator proteins of the IRG system, and probably results from the cytopathic effects of activated GKS subfamily IRG protein aggregates in the cytoplasm (Hunn *et al.*, 2008; Papic *et al.*, 2008; Henry *et al.*, 2009a). This complex conclusion shows how important it is to study the interacting components of the IRG system in order to understand the implications of single IRG gene knockout data. Resistance to *T. gondii* does not follow the Irgm1 paradigm as the Irgm1/Irgm3 doubly deficient mouse is highly susceptible to the pathogen (Henry *et al.*, 2009a). Thus resistance to *T. gondii* (and presumably also *C. trachomatis*) is truly dependent on the integrity of the IRG system. An issue for the future is to understand what the *T. gondii* and *C. trachomatis* vacuoles have in common that makes them targets for IRG protein action.

## Experimental procedures

### Expression constructs

The following mammalian expression constructs were used: pEGFP-N3-Irga6-ctag1, pmCherry-N3 (both from Zhao *et al.*, 2009a); pGW1H-Irgm1 (Martens *et al.*, 2004); pGW1H-Irgb6-FLAG, pGW1H-Irgm2, pGW1H-Irgm3 and pGW1H-Irgd (all from Hunn *et al.*, 2008). pmDsRed-N3-Irgb6-FLAG was made via PCR amplification of Irgb6-FLAG from pGW1H-Irgb6-FLAG construct using the following primers: forward 5′-cccccccccgtcgaccaccatggcttgggcctccagc-3′ and reverse 5′-cccccccccgtcgaccttgtcatcgtcgtccttgtaatc-3′ and insertion into pmDsRed-N3 (Zhao *et al.*, 2009a) following SalI digestion. pEGFP-N3-Irgb6-FLAG was generated by subcloning Irgb6-FLAG fragment from pmDsRed-N3-Irgb6-FLAG into pEGFP-N3 (Clontech) using SalI digestion. pmCherry-N3-Irgd-ctag1 was generated by PCR amplification of Irgd-ctag1 from pGW1H-Irgd-ctag1 (Hunn *et al.*, 2008) using the following primers: forward 5′-ccccccgtcgaccaccatggatcagttcatctcagcc-3′ and reverse 5′-ccccccgtcgacgtcacgatgcggccgctcgagtcgg-3′ and by cloning it into pmCherry-N3. pGW1H-unROP18 ME49 and pGW1H-unROP18 RH-YFP containing the unprocessed forms of ROP18 were generated by PCR amplification from genomic DNA of ME49 and RH-YFP *T. gondii* strains using the following primers: forward 5′-ccccccgtcgaccaccatgttttcggtacagcggcc-3′ and reverse 5′-ccccccgtcgacttagtcaagtggatcctggttagtatggacctcttctgtgtggagatgttcctgc-3′ and subsequent cloning into the SalI site of pGW1H (British Biotech). The C-terminally Ty-tagged mature forms of ROP18 were amplified from pGW1H-unROP18 ME49 and pGW1H-unROP18 RH-YFP using the following primers: forward 5′-ccccccgtcgaccaccatggaaagggctcaacaccgggta-3′ and reverse 5′-ccccccgtcgacttagtcaagtggatcctggttagtatggacctcttctgtgtggagatgttcctgc-3′ and cloned into pGW1H by SalI digestion. Pfu-polymerase (Promega) was used for PCR amplification and primers were from Operon Biotechnologies GmbH. Restriction enzymes were from New England Biolabs. All constructs were verified by sequencing.

### Cell culture

gs3T3 cells (Invitrogen), Mifepristone-inducible gs3T3-Irga6 cells (Hunn *et al.*, 2008), A31 3T3 cells (ATCC, CCl-163), Atg5^−/−^ and the corresponding wt control immortalized mouse fibroblasts (Kuma *et al.*, 2004, kindly provided by Martin Krönke and Nobura Mizushima), MyD88^−/−^ MEFs (Adachi *et al.*, 1998, kindly provided by Manolis Pasparakis), Irga6^−/−^ MEFs (Martens *et al.*, 2005), L929 cells (ATCC CCl-1), HFF cells (ATCC, CRl-1634) and C57BL/6 MEFs were cultured in DMEM, high glucose (Invitrogen) supplemented with 10% fetal calf serum (FCS) (Biochrom), 2 mM l-glutamine, 1 mM sodium pyruvate, 1× MEM non-essential amino acids, 100 U ml^−1^ penicillin and 100 mg ml^−1^ streptomycin (all PAA). Transient transfection of mouse fibroblasts was conducted using FuGENE6 (Roche) according to the manufacturer's instructions. Cells were induced with 200 U ml^−1^ of mouse IFNγ (Peprotech) or 10^−9^M Mifepristone (Invitrogen) for 24 h.

To analyse the influence of other IRG proteins on the loading of Irga6 onto PVs of *T. gondii* strain ME49 pools of pGW1H-IRG expression constructs were transiently transfected into Mifepristone-induced gs3T3-Irga6 cells (Hunn *et al.*, 2008) grown on coverslips in six-well plates. In all cases 400 ng of each construct were co-transfected in a total amount 2 µg of DNA. Empty vector was used to adjust DNA amounts if less than five constructs were transfected.

### Immunological reagents

The following immunoreagents were used: rabbit anti-Irga6 antiserum 165/3 (Martens *et al.*, 2004), mouse anti-Irga6 monoclonal antibodies (mAb) 10E7 and 10D7 (Papic *et al.*, 2008), mouse anti-Irgm3 monoclonal antibody anti-IGTP (BD Biosciences, 610881), goat anti-Irgb6 antiserum A20 (Santa Cruz Biotechnology, sc-11079), rabbit anti-Irgb6 antiserum 141/1 was raised against recombinant bacterial full-length protein (N. Pawlowski, unpublished data), mouse anti-Irgb6 monoclonal antibody B34 (Carlow *et al.*, 1998), rabbit anti-Irgm2 antiserum H53/3, raised against the N-terminal peptide of Irgm2 from C57BL/6 and unreactive against Irgm2 from 129 strain mice (Martens *et al.*, 2005 and J.C. Howard, unpublished data), rabbit anti-Irgd antiserum 2078/3 (Martens *et al.*, 2004), rabbit anti-Irgd antiserum 081/1 was raised against bacterially synthesized full-length protein (N. Pawlowski and G. Vopper, unpublished data), rabbit anti-Irgb10 antiserum (Coers *et al.*, 2008), anti-Irgm1 antiserum L115 B0 (Martens *et al.*, 2005), anti-*T. gondii* rabbit (BioGenex, PU125-UPE) and goat antisera (Abcam, ab23507), mouse IgG anti-GRA7 monoclonal antibody (Bonhomme *et al.*, 1998) and mouse IgM anti-GRA7 monoclonal antibody TxE2 (Fischer *et al.*, 1998), rabbit anti-ctag1 antiserum 2600 (Martens *et al.*, 2005), rabbit anti-calnexin antiserum (StressGene, SPA-865), mouse anti-α-tubulin monoclonal antibody (Sigma-Aldrich, T 6074), mouse anti-Ty-tag monoclonal antibody (Bastin *et al.*, 1996, kindly provided by Keith Gull), rat anti-LAMP1 monoclonal antibody (University of Iowa), anti-Akt (Cell Signaling, 9272) and anti-phosphoAkt (Cell Signaling, 9271) rabbit polyclonal antibodies, rabbit anti-PARP1 polyclonal antibody (Cell Signaling, 9542), Alexa 350/488/546/555/647-labelled donkey anti-mouse, rabbit and goat sera (Molecular Probes), donkey anti-rabbit- (GE Healthcare), donkey anti-goat- (Santa Cruz Biotechnology) and goat anti-mouse-HRP (horseradish peroxidase) (Pierce) antisera.

The serum 081/1, used to identify Irgd, produced occasional signals in IFNγ-stimulated Irgd^−/−^ MEFs (S. Könen-Waisman, unpublished data). These signals were extremely faint compared with the strong signals from wt cells measured both by Western blot and immunofluorescence. Considering the significant homology between the IRG proteins we assume that the reagent is cross-reacting weakly on another family member. Nevertheless the counted frequency of positive vacuoles found with 081/1 corresponded with that seen with rabbit antiserum 2078/3 (Martens *et al.*, 2004), a much weaker anti-peptide antiserum, used at high concentration.

### Inhibition of signalling pathways and microtubule polymerization

To block PI3 kinase and G protein-coupled receptors overnight FCS-starved C57BL/6 MEFs were pretreated with wortmannin (0.5 µM), LY294002 (2-(4-morpholinyl)-8-phenyl-4H-1-benzopyran-4-one) (25 µM) and pertussis toxin (200 ng ml^−1^) for 6 h (all reagents were derived from Sigma-Aldrich and handled according to the manufacturers protocol). The extent of inhibition was tested by monitoring in Western blot the level of phospho-Akt (pAkt) after 10 min stimulation with EGF (epidermal growth factor) (100 ng ml^−1^) (Peprotech). To block caspase activity MEFs were pretreated with z-VAD-fmk (benzyloxycarbonyl-Val-Ala-Asp fluoromethyl ketone) pan-caspase inhibitor (100 µM) (Alexis Biochemicals, 260-020-M005) for 2 h, and the degree of blockade was analysed by monitoring the processing of PARP1 (poly-ADP ribose polymerase 1) 6 h after TNFα (40 ng ml^−1^) (Peprotech) plus cycloheximide (Chx) (10 µg ml^−1^) stimulation in Western blot. Inhibition of microtubule polymerization was achieved by incubating MEFs in 10 µM nocodazole in DMSO (Sigma-Aldrich) for 1 h and was monitored microscopically after performing immunostaining using anti-α-tubulin mouse monoclonal antibody.

To assay IRG protein association with the PV under conditions of blocked signalling pathways or inhibited microtubule polymerization, IFNγ-stimulated MEFs were pretreated as described above or left untreated as a control, then infected with ME49 *T. gondii* for 2 h and stained for Irgb6 (serum A20) and Irga6 (mAb 10D7). To study MyD88 involvement in PV loading by IRG proteins IFNγ-stimulated MyD88^−/−^ and wt MEFs were infected with ME49 *T. gondii* and stained for Irgb6 (serum A20) and Irga6 (mAb 10D7).

### Passaging of *T. gondii* and infection of murine fibroblasts

The following *T. gondii* strains were used: type I virulent RH (Lecomte *et al.*, 1992), RH-Δrop16 [in which the ROP16 locus has been deleted through double homologous recombination using HXGPRT for selection and PCR for confirmation of the deletion, as previously described (Saeij *et al.*, 2008)], RH-YFP (Gubbels *et al.*, 2003) and BK (Winsser *et al.*, 1948); type II avirulent ME49 (Guo *et al.*, 1997), NTE (Gross *et al.*, 1991), avirulent recombinant *T. gondii* strains S22 (Saeij *et al.*, 2006) and S22-LC37, the latter harbouring a cosmid containing four ROP5 genes (ROP5A–D) along with two adjacent genes (annotated gene models TGME49_108070 and TGME49_108060) from the RH strain and introduced using bleomycin selection; type III avirulent CTG strain (Pfefferkorn and Kasper, 1983, kindly provided by Dominique Soldati-Favre). Tachyzoites of different *T. gondii* strains were passaged in HS27 cells (Martens *et al.*, 2005), and used for infection of untreated, transiently transfected, IFNγ- and/or Mifepristone-induced fibroblasts at a multiplicity of infection (moi) from 5 to 10 as described previously (Martens *et al.*, 2005). *Toxoplasma* infection of MEFs was synchronized according to Kafsack *et al.* (2004; 2007;). In brief, the parasites were resuspended to 5 × 10^6^ parasites per millilitre in invasion non-permissive Endo buffer (44.7 mM K_2_SO_4_, 10 mM MgSO_4_, 106 mM sucrose, 5 mM glucose, 20 mM Tris–H_2_SO_4_, 3.5 mg ml^−1^ BSA, pH 8.2). One millilitre of tachyzoite suspension was added to each well on the six-well plate, the plates centrifuged at 1500 r.p.m. for 2 min and then placed in the incubator. The infection was synchronized by replacing the Endo buffer by permissive medium (IMDM + 10 mM Hepes buffer, pH 7.4, and 5% FCS) for 2 min. Free parasites were subsequently removed by repeated washing with medium until no free parasites could be detected microscopically. At various times after infection, cells were washed with PBS and fixed in PBS/3% paraformaldehyde. Non-synchronized cells were handled throughout in IMDM/5% FCS, parasites were added at time zero, and the cells were examined at various times thereafter after washing and fixation.

### *T. gondii* proliferation assay

The growth of *T. gondii* was analysed using the ^3^H-uracil incorporation assay (Pfefferkorn and Guyre, 1984). IFNγ-induced MEFs were infected with specified *T. gondii* strains for 24 h at moi 0.3, 1 and 3. The cultures were labelled with 0.3 µCi well^−1^ of ^3^H-uracil (^3^HU, Harmann Analytic) for 24 h and then frozen at −20°C. The amount of radioactivity incorporated into the proliferating parasites was determined in a β-scintillation spectrometer. The data are shown either as radioactive counts (Fig. S7), which are proportional to the parasite growth, or as the percentage of parasite growth inhibition caused by IFNγ treatment (Fig. 9C). Growth inhibition was defined as follows:


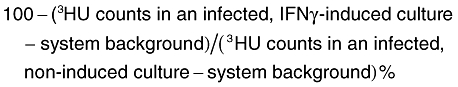


where system background is ^3^HU counts in an uninfected, non-induced culture.

### Immunocytochemistry

Immunocytochemistry was performed on paraformaldehyde-fixed cells as described earlier (Martens *et al.*, 2004; 2005;); the images were taken with an Axioplan II fluorescence microscope and AxioCam MRm camera and processed by Axiovision 4.7 (all Zeiss) and Image J softwares (http://rsb.info.nih.gov/ij/). 4′,6-Diamidine-2′-phenylindole dihydrochloride (DAPI, Invitrogen) was used for nuclear counterstaining at a final concentration of 0.5 µg ml^−1^. Intracellular parasites were identified by observing the vacuolar localization of the *T. gondii* protein GRA7 or by distinct pathogen appearance in phase contrast.

### Live cell imaging

Live cell imaging was performed as described earlier (Zhao *et al.*, 2009a).

### Quantification of IRG protein signal intensity at *T. gondii* PV

The measurements were performed using the Image J (http://rsb.info.nih.gov/ij/) and Axiovision 4.7 (Zeiss) software. To measure the intensity of fluorescent signal on a labelled PVM, two lines were drawn at right angles across the long and short axes of the vacuole, and pixel intensity profiles obtained for each line (Fig. S1). The first and last values for each line provided four estimates of ‘background’ signal, while the four peaks where each line crossed the margins of the PVM gave four independent values for the signal strength at the vacuole. The signal intensity for the vacuole was given as the mean of the four peak values minus the mean of the four background values.

### SDS-PAGE and Western blot

Cells were lysed in 1% Triton X-100/PBS/Complete Mini Protease Inhibitor Cocktail, EDTA-free (Roche) or 1× sample buffer (80 mM Tris-HCI, 5 mM EDTA, 34% Sucrose, 3.2% SDS, 40 mM DTT, bromophenol blue). Post-nuclear supernatants were subjected to SDS-PAGE and Western blot. Membranes were probed for IRG proteins with the indicated primary and HRP-coupled secondary antibodies for chemiluminescence.

### Statistical analysis of data

For those experimental paradigms (Figs 6A and 8B), for which the number of experiments was large enough, significance was assessed with a modified Student's *t*-test after Dixon and Massey (Dixon and Massey, 1969). This *t*-test allows the comparison of sample sizes less than 30 and with unequal variances. For those experimental paradigms for which the number of experiments did not exceed *n* = 3 (Figs 5A and 8A), original data from the individual experiments were pooled and compared with a chi-squared test (Greenwood and Nikulin, 1996). Sample parameters were considered to be significantly different throughout this study with *P* < 0.05. The significances of differences between pixel intensity distributions shown in Figs 5B, 6F, 7 and 8D were estimated by the Mann–Whitney *U*-test (Mann and Whitney, 1947).

## Supporting information

**Fig. S1.** Analysis of IRG pixel intensity on *T. gondii* PVs.

Details of this analysis are given in *Experimental procedures*. To measure the intensity of IRG signal on *T. gondii* PVs two lines were drawn approximately at right angles across each vacuole (A). Each line thus crossed the ‘margin’ of the vacuole twice, giving a total of four values for each vacuole using the Image J and Axiovision 4.7 software. The pixel intensity of each vacuole was defined as the mean of these four values after background subtraction (B). To investigate how much variation in signal intensity measurements is caused by inhomogeneity of the IRG signal around the rim of the PV, 20 profiles were created by drawing 20 parallel lines through each of three more or less brightly Irgb6- positive vacuoles (stained by serum A20). The mean values of pixel intensity were calculated from each profile and presented as single dots and the arithmetic means are given as horizontal lines (C). Variation of Irgb6 intensity around the rim of single PVs clearly does not account for the total heterogeneity of IRG protein signal shown on Fig. 2A and B and elsewhere as the different signal intensities on the three vacuoles can be clearly distinguished. (D) Selected frames of two time-lapse videos of Irgb6- FLAG-EGFP loading on ME49 *T. gondii* PV. MEFs were transfected with the expression plasmid pEGFP-N3-Irgb6-FLAG and simultaneously induced with IFNγ. After 24 h, the cells were infected with *T. gondii* ME49 strain in microscope slide chambers as described in Zhao et al. (2009a) and monitored continuously in order to document the entry of individual parasites and the subsequent accumulation of Irgb6-FLAG-EGFP on the PV. Arrowheads indicate the location of the analysed *T. gondii* PVs.

**Fig. S2.** Inhibition of major signalling pathways and microtubule polymerization.

A. The blockade of PI3 kinase and G protein-coupled receptors was demonstrated by Western blot for phospho-Akt (pAkt). MEFs were treated with wortmannin (W), LY294002 (LY) or pertussis toxin (PT) for 6 h as described in *Experimental procedures*. Inhibited cells were stimulated by EGF for 10 min to induce Akt phosphorylation. Calnexin and total Akt served as a loading control. B. To inhibit caspases MEFs were treated with z-VAD-fmk for 2 h followed by stimulation with TNFα for 6 h in the presence of cycloheximide (Chx), as described in *Experimental procedures*. The amount of PARP1 processing by caspases was analysed by Western blot. Calnexin served as a loading control.

C. Inhibition of microtubule polymerization was monitored microscopically by immunostaining with an anti-α-tubulin mouse monoclonal antibody. MEFs were induced with IFNγ for 24 h and treated with nocodazole, or DMSO as control, for 1 h, as described in *Experimental procedures*. Treated cells were infected with *T. gondii* ME49 strain and loading of parasite PVs was monitored 2 h after infection by immunostaining for Irgb6 (serum A20).

**Fig. S3.** Irga6 aggregates do not colocalize with lysosomal marker LAMP1 in Atg5^-/-^ fibroblasts. Cells were prepared as described in Fig. 5A and immunostained for Irga6 (mAb 10D7) and LAMP1 (mAb 1D4B). No obvious colocalization of Irga6 with LAMP1 in wt and Atg5^-/-^ fibroblasts was observed. Arrows indicate intracellular parasites identified by phase contrast (PhC), and arrowheads indicate Irga6 aggregates.

**Fig. S4.** Frequencies of IRG protein-positive vacuoles detected by specific antibody reagents at different dilutions: saturation of frequency estimates. IFNγ-induced C57BL/6 MEFs were infected with *T. gondii* ME49 for 2 h. IRG proteins were detected by serial twofold dilution of antibody reagents around the dilutions typically used for experiments followed by an appropriate secondary reagent to estimate loaded vacuole frequencies. The experimental dilutions for each reagent were as follows: for Irgb6: mAb B34 at 1/1000 (3.4 mg ml^-1^ stock), serum A20 at 1/200; for Irgb10: serum anti-Irgb10 at 1/2000; for Irga6: mAb 10E7 at 1/1000 (3.7 mg ml^-1^ stock), mAb 10D7 at 1/2000 (4.3 mg ml^-1^ stock), serum 165/3 at 1/8000; for Irgd: serum 081/1 at 1/4000. The most concentrated dilution is designated as 1 (mAb B34 at 1:250, serum A20 at 1:50, serum anti-Irgb10 at 1:500, mAb 10E7 at 1:100, mAb 10D7 at 1:500, serum 165 at 1:2000, serum 081/1 at 1:4000). IRG-positive PVs were counted blind from 100–200 intracellular parasites. Frequency estimates for loaded vacuoles were independent of antibody or antiserum dilution in the range tested.

**Fig. S5.** Irga6-ctag1-EGFP loads before Irgd-ctag1-Cherry onto *T. gondii* ME49 strain PV. C57BL/6 MEFs were induced with IFNγ and transfected simultaneously with constructs expressing Irga6- ctag1-EGFP and Irgd-ctag1-Cherry. After 24 h, cells were infected with *T. gondii* ME49 strain in microscope slide chambers and observed by live cell imaging for the accumulation of IRG proteins. Successive 3 min frames from one vacuole (arrow) show Irga6 visibly loading several frames before Irgd.

**Fig. S6.** Irga6 and Irgb6 pixel intensity profiles of RH-YFP PVs in IFNγ-induced cells. IFNg-induced MEFs were infected with the *T. gondii* strain RH-YFP for 2 h. The pixel intensities of the Irga6 (mAb 10D7, red) and serum Irgb6 (A20, blue) signals at the RH-YFP (green) PVM across transects of the vacuole are displayed as a function of distance (μm). Five representative PVs of the three categories Irga6 and Irgb6 double-positive (A), Irga6 single-positive (B) and double-negative (C) are given (see also Fig. 8E). The baseline of the A20 serum staining is high due to diffuse background staining of the blue second stage reagent.

**Fig. S7.** IFNγ-restrained proliferation of *T. gondii* is not affected by the virulence-associated ROP proteins, ROP5 and ROP16. The growth of RH-Δrop16, S22-LC37 (carrying the cosmid expressing 4 ROP5 and 2 other genes) and control RH and S22 *T. gondii* strains was measured by ^3^H-uracil incorporation as described in *Experimental procedures*. The data are presented in radioactive counts per 3 min. Black, light grey and dark grey bars indicate proliferation of the parasite in MEFs untreated, stimulated with 10 U ml^-1^ and 100 U ml^-1^ of IFNγ respectively.

**Table S1.** Summary of experiments analysing the correlated loading of selected pairs of IRG proteins on to the ME49 PV.

**Video S1.** Time-lapse video of Irga6-ctag1-EGFP association with the *T. gondii* PV. IFNγ-treated MEFs were transfected with the construct expressing Irga6-ctag1-EGFP and infected with *T. gondii* ME49. Time-lapse video was started immediately after infection and the images were collected every 30 s (see also Fig. 2A). The video is presented at 6 frames per second.

**Video S2.** Time-lapse video of Irga6-ctag1-EGFP association with the *T. gondii* PV. IFNγ-treated MEFs were transfected with the construct expressing Irga6-ctag1-EGFP and infected with *T. gondii* ME49. Time-lapse video was started 1 h after infection and the images were collected every 3 min (see also Fig. 2B). The video is presented at two frames per second. Out of focus frames were excluded from the movie.
