# Supplementary figures and images for: Coordinated loading of IRG resistance GTPases on to the Toxoplasma gondii parasitophorous vacuole
Source: Cell Microbiol. 2010 Mar 4;12(7):939–61. doi: 10.1111/j.1462-5822.2010.01443.x (PMC2901525; doi:10.1111/j.1462-5822.2010.01443.x)

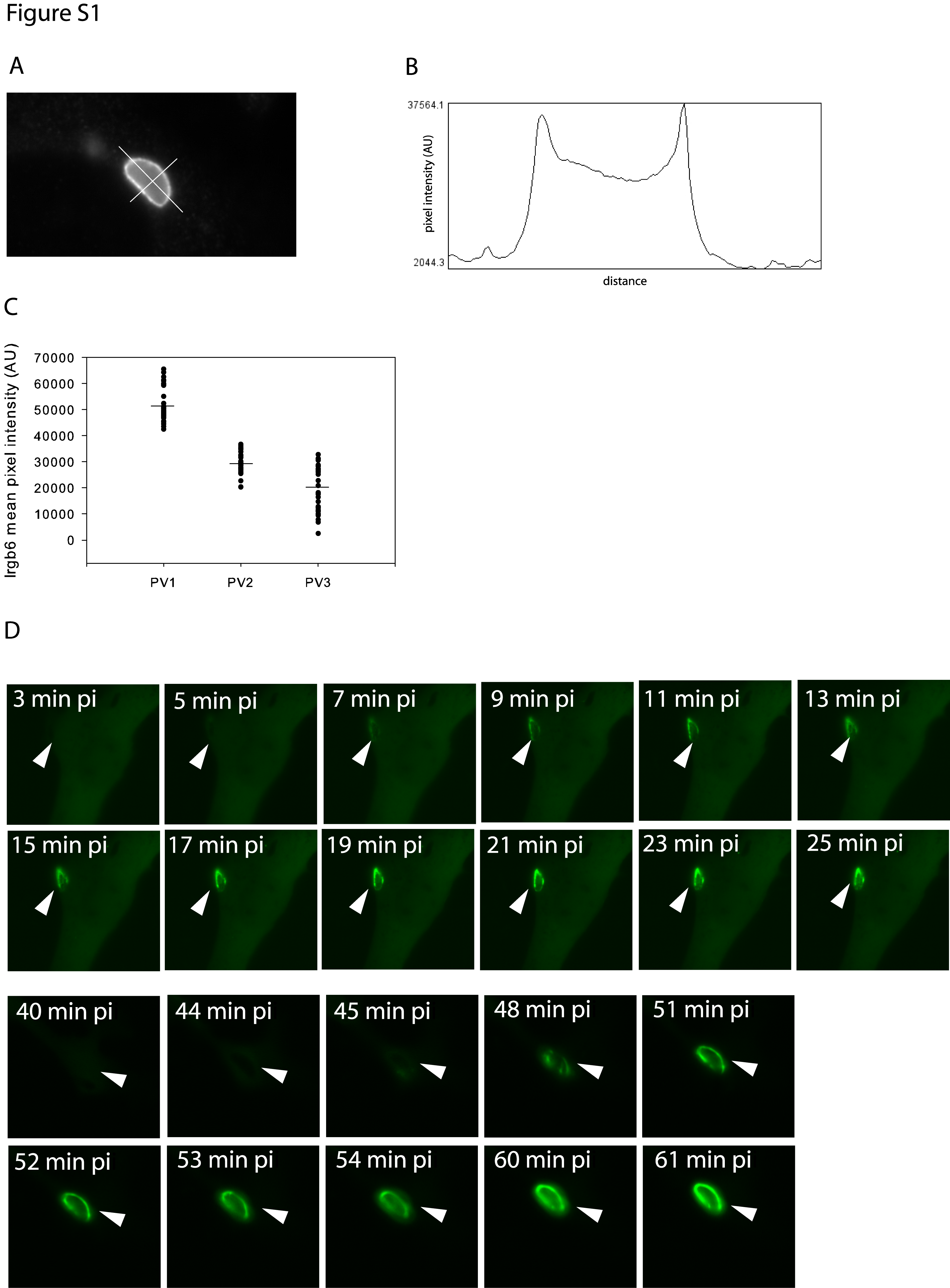

Supplement: Supplementary file 1 [file cmi0012-0939-SD1.tif]

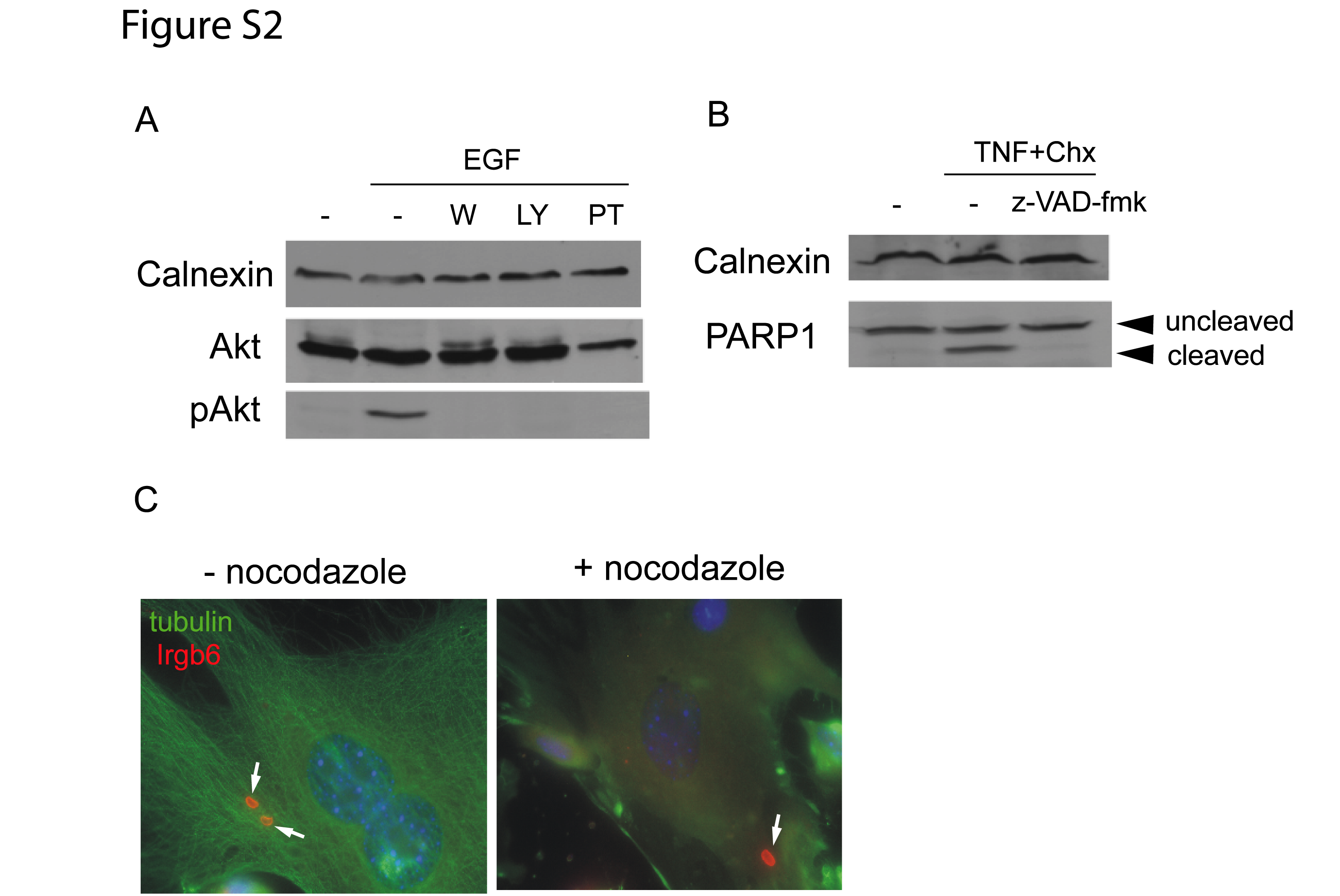

Supplement: Supplementary file 2 [file cmi0012-0939-SD2.tif]

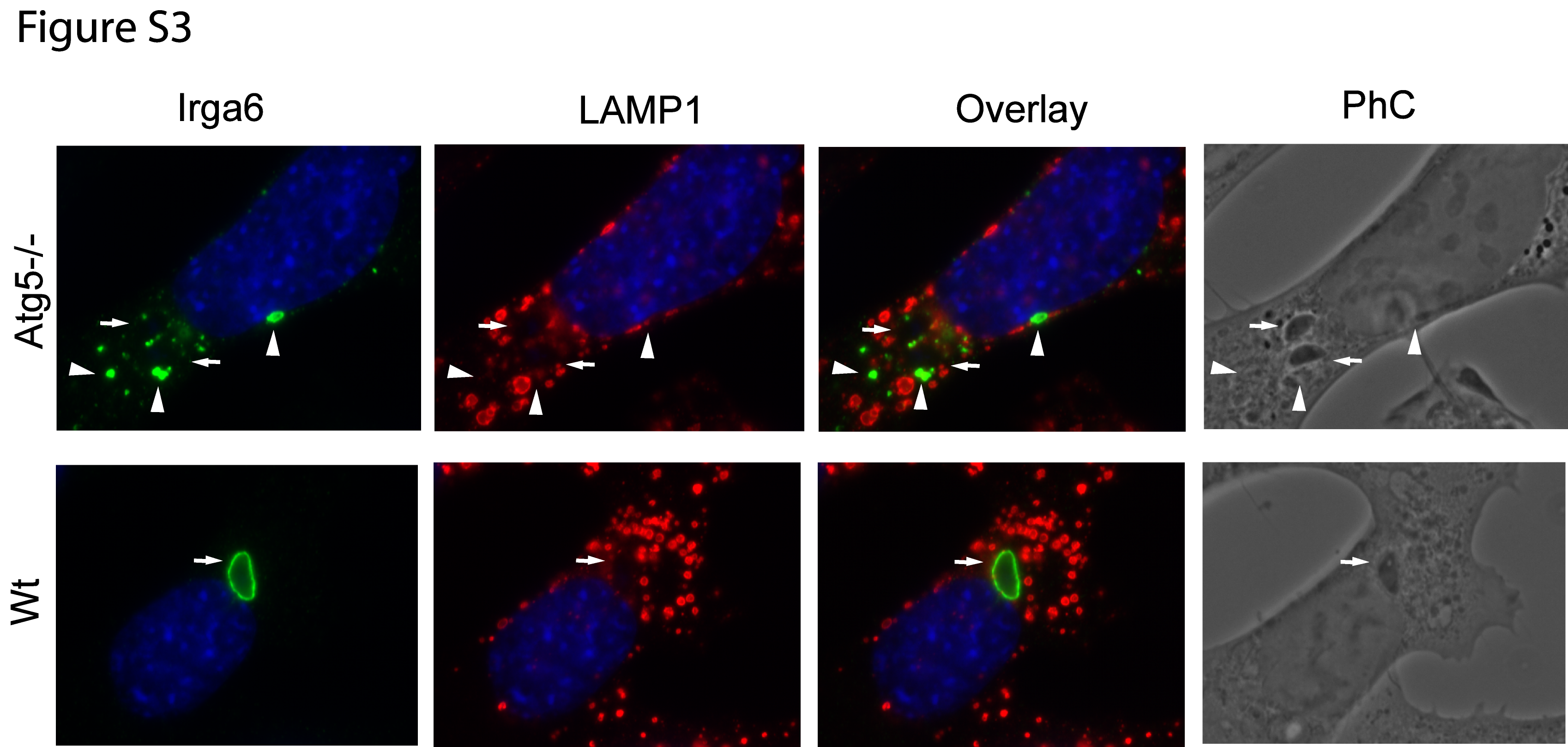

Supplement: Supplementary file 3 [file cmi0012-0939-SD3.tif]

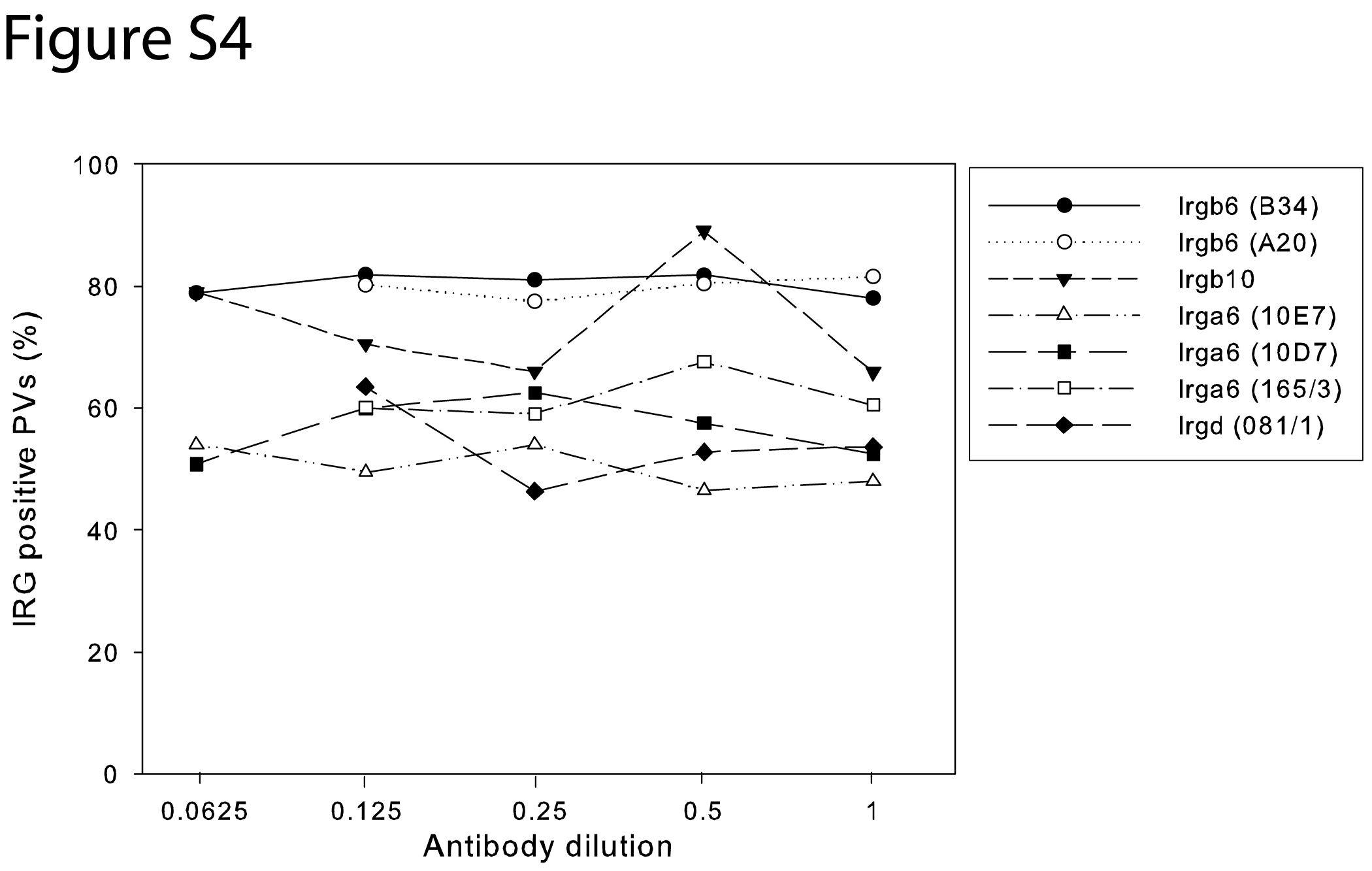

Supplement: Supplementary file 4 [file cmi0012-0939-SD4.tif]

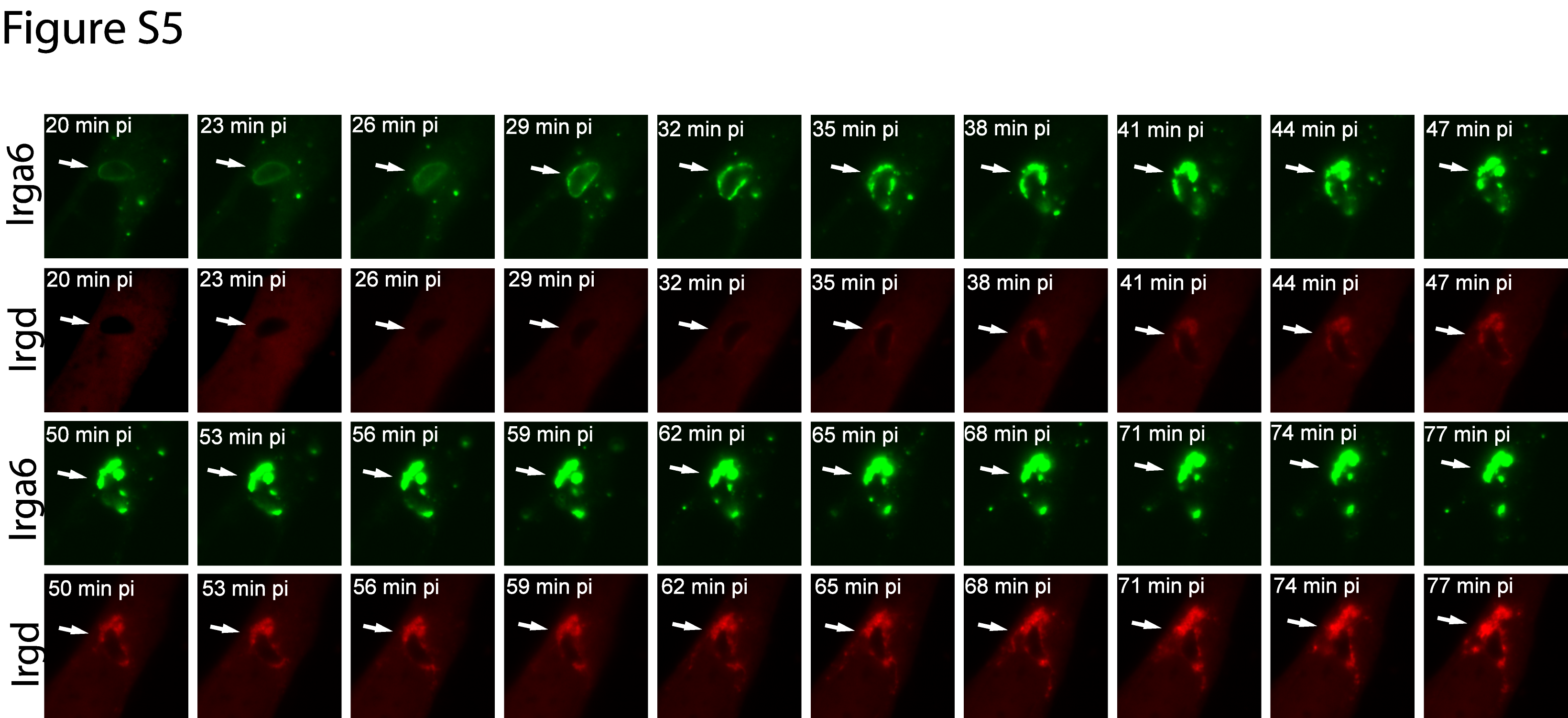

Supplement: Supplementary file 5 [file cmi0012-0939-SD5.tif]

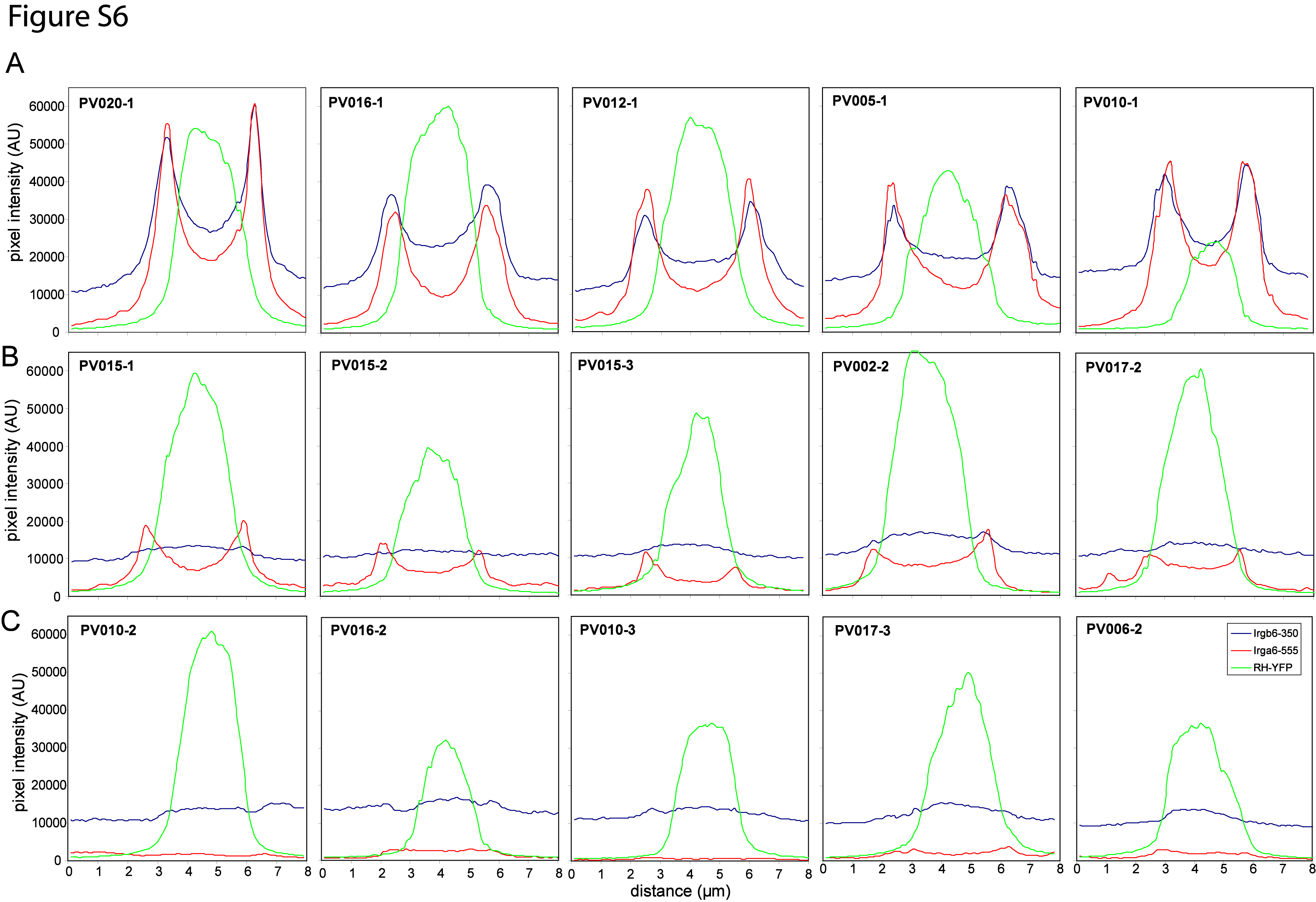

Supplement: Supplementary file 6 [file cmi0012-0939-SD6.tif]

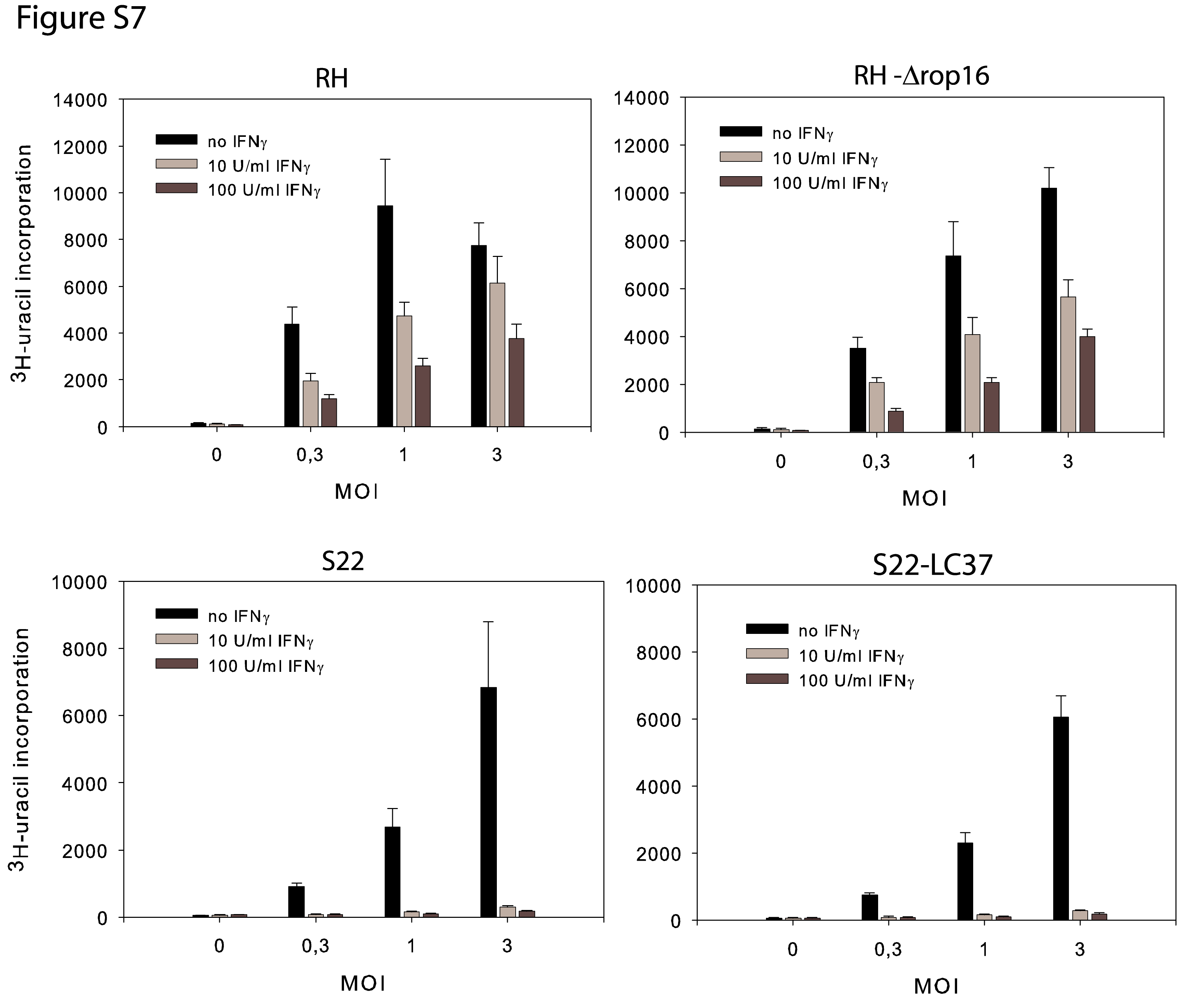

Supplement: Supplementary file 7 [file cmi0012-0939-SD7.tif]
