# Supplementary material for: Coordinated loading of IRG resistance GTPases on to the Toxoplasma gondii parasitophorous vacuole
Source: Cell Microbiol. 2010 Mar 4;12(7):939–61. doi: 10.1111/j.1462-5822.2010.01443.x (PMC2901525; doi:10.1111/j.1462-5822.2010.01443.x)
Supplement: Supplementary file 8 [file cmi0012-0939-SD8.doc]

Table S1. Summary of experiments analysing the correlated loading of selected pairs of IRG proteins on to the ME49 PV.

| A versus B |  | Only A | A+B | Only B | Total |
| --- | --- | --- | --- | --- | --- |
| Irgb6 *vs* Irgb10 | IRG positive PVs | 159 | 1142 | 130 | 1431 |
|  | % of total PVs | 11.1% | 79.8% | 9.1% |  |
|  |  |  |  |  |  |
| Irgb6 *vs* Irga6 | IRG positive PVs | 212 | 612 | 10 | 834 |
|  | % of total PVs | 25.4% | 73.3% | 1.2% |  |
|  |  |  |  |  |  |
| Irgb6 *vs* Irgd | IRG positive PVs | 419 | 283 | 1 | 703 |
|  | % of total PVs | 59.55% | 40.25% | 0.14% |  |
|  |  |  |  |  |  |
| Irgb6 *vs* Irgm2 | IRG positive PVs | 298 | 310 | 1 | 609 |
|  | % of total PVs | 48.9% | 50.9% | 0.1% |  |
|  |  |  |  |  |  |
| Irgb10 *vs* Irga6 | IRG positive PVs | 91 | 216 | 11 | 318 |
|  | % of total PVs | 28.6% | 67.9% | 3.5% |  |
|  |  |  |  |  |  |
| Irga6 *vs* Irgd | IRG positive PVs | 327 | 390 | 0 | 718 |
|  | % of total PVs | 45.5% | 54.3% | 0.14% |  |
|  |  |  |  |  |  |
| Irga6 *vs* Irgm2 | IRG positive PVs | 36 | 113 | 0 | 149 |
|  | % of total PVs | 24.2% | 75.8% | 0 |  |
|  |  |  |  |  |  |
| Irga6 *vs* Irgm3 | IRG positive PVs | 10 | 101 | 0 | 111 |
|  | % of total PVs | 9.0% | 91.0% | 0 |  |

IFNγ stimulated mouse fibroblasts were infected with *T. gondii* ME49 for 2h and doubly or triply co-stained for distinct IRG proteins using appropriate immunoreagents as described in Fig. S4 and Materials and Methods. Vacuoles positive for any IRG protein were analysed for the presence of the other protein of the family and single and double IRG protein positive PVs were quantified. The data are presented with the first and second IRG protein designated as A and B respectively. (Only A), (Only B) and (A+B) indicate number of vacuoles positive for only first, only second or positive for both IRG proteins. At least 100 individual vacuoles were scored. Vacuoles loaded with neither IRG protein were not included in the analyses. See also Fig. 6B. These results establish that the IRG proteins not only load on to more or fewer vacuoles, but that loading is correlated to yield an “inclusion” or “Russian Doll” relationship between the proteins.
